# The transmembrane domain of the amyloid precursor protein is required for antiamyloidogenic processing by α-secretase ADAM10

**DOI:** 10.1016/j.jbc.2022.101911

**Published:** 2022-04-07

**Authors:** Lisa Hitschler, Thorsten Lang

**Affiliations:** Department of Membrane Biochemistry, Life & Medical Sciences (LIMES) Institute, University of Bonn, Bonn, Germany

**Keywords:** ADAM17, secretases, Alzheimer's disease, amyloid beta, super-resolution microscopy, Aβ, amyloid β-peptide, AD, Alzheimer's disease, ADAM, a disintegrin and metalloproteinase, APP, amyloid precursor protein, BSA, bovine serum albumin, CoP, coprecipitated, CTF, C-terminal APP fragment, DAPT, *N*-[*N*-(3,5-difluorophenacetyl)-l-alanyl]-S-phenylglycine *t*-butyl ester, EGFR, epidermal growth factor receptor, GFP, green fluorescent protein, IF, immunofluorescence, PCC, Pearson correlation coefficient, PFA, paraformaldehyde, PMA, phorbol 12-myristate 13-acetate, ROI, region of interest, RT, room temperature, rSDM, relative standard deviation of the mean, sAPPα, soluble APPα, STED, stimulated emission depletion, TMS, transmembrane segment, WB, Western blot

## Abstract

Neurotoxic amyloid β-peptides are thought to be a causative agent of Alzheimer’s disease in humans. The production of amyloid β-peptides from amyloid precursor protein (APP) could be diminished by enhancing α-processing; however, the physical interactions between APP and α-secretases are not well understood. In this study, we employed super-resolution light microscopy to examine in cell-free plasma membranes the abundance and association of APP and α-secretases ADAM10 (a disintegrin and metalloproteinase) and ADAM17. We found that both secretase molecules localize similarly closely to APP (within ≤50 nm). However, when cross-linking APP with antibodies directed against the GFP tag of APP, in confocal microscopy, we observed that only ADAM10 coaggregated with APP. Furthermore, we mapped the involved protein domain by using APP variants with an exchanged transmembrane segment or lacking cytoplasmic/extracellular domains. We identified that the transmembrane domain of APP is required for association with α-secretases and, as analyzed by Western blot, for α-processing. We propose that the transmembrane domain of APP interacts either directly or indirectly with ADAM10, but not with ADAM17, explaining the dominant role of ADAM10 in α-processing of APP. Further understanding of this interaction may facilitate the development of a therapeutic strategy based on promoting APP cleavage by α-secretases.

Alzheimer's disease (AD) is the most prevalent form of dementia. In the United States, 6.2 million Americans with an age of 65 or older are living with AD (1), an incidence expected to rise further simply because of an aging population (2).

The disease is characterized by extracellular plaques in the brain consisting of neurotoxic amyloid β-peptides (Aβ) and intraneuronal hyperphosphorylated Tau protein aggregates (3).

The source of Aβ is the amyloid precursor protein (APP), from which Aβ is produced by consecutive proteolysis of the β- and γ-cleavage sites (4). β-site cleavage defines the N-terminus of the Aβ region. It produces two fragments, a large APP ectodomain (soluble APPβ) that is secreted and a C-terminal APP fragment (β-CTF) in the membrane. β-CTF is further processed by the γ-secretase complex (5) yielding Aβ peptides that eventually are released into the extracellular space, where they deposit into plaques. This describes the unfavorable so-called amyloidogenic pathway. It can be avoided by initial cleavage by α-secretases cutting downstream of the β-site between amino acids 16 and 17 of the Aβ region (6, 7). Thus, α-cleavage yields a longer ectodomain (soluble APPα; sAPPα) and a shorter C-terminal fragment (α-CTF). Because α-CTF is shorter, γ-cleavage produces shorter nonaggregating and non-neurotoxic peptides, referred to as the antiamyloidogenic pathway (4).

A therapeutic strategy for AD treatment is to shift APP processing towards the antiamyloidogenic pathway by β-secretase inhibition, which should boost α-processing. The strategy has been explored for many years, but in the meantime, it has been considered to have failed because of too many side effects (8, 9), which could be due to the diversity in β-secretase substrates (10).

One interesting aspect is that α- and β-secretases are active at different cellular locations; β-cleavage occurs primarily intracellularly in the endosomal system (11, 12). Consequently, endocytosis of APP is a prerequisite for amyloidogenic processing (12). On the other hand, α-processing takes place almost exclusively at the cell surface, suggested by ∼90% inhibition of α-cleavage by a cell-impermeable inhibitor (13). An alternative to the inhibition of β-secretase would be increasing α-secretase processing prior to endocytosis (14). This seems feasible because α-secretase overexpression in mice expressing human APP increases sAPPα levels and reduces the formation of Aβ peptides and plaques (15). This finding indicates that α- and β-secretases indeed compete for APP as a substrate (15, 16) and have opposite effects on Aβ generation (15).

While β-secretase activity can be clearly assigned to BACE1 (β-site APP cleaving enzyme) (17), the picture is more complex regarding α-secretases. APP is processed by several members of the ADAM (a disintegrin and metalloproteinase) family, including ADAM9, ADAM10, ADAM17/TACE, and ADAM19 (18, 19, 20, 21). Moreover, ADAMs are subject to the regulation through protein kinase C.

Studies in which ADAM10 is overexpressed, knocked down, mutated, or its trafficking to the cell membrane is increased (15, 19, 22, 23, 24, 25) indicate that ADAM10 is the main secretase responsible for constitutive α-processing. For instance, knockdown of ADAM10 in neuronal cell lines and primary cortical neurons, as well as conditional knockdown in mice, strongly reduces sAPPα by 79 to 90% (22, 23, 24). Regarding ADAM9 and ADAM17, phorbol ester–stimulated cleavage is observed (18, 20, 26, 27). Stimulated increase of sAPPα is completely abolished in ADAM17 knockout mice, indicating that ADAM17 is responsible for the majority of regulated α-cleavage (18).

Understanding on a molecular level why ADAM10 is more active in α-processing than other secretases would be helpful in developing a strategy for reducing Aβ production. However, the molecular details of the enzyme–substrate interaction are not yet understood.

In this study, we characterize the association of APP and the secretases ADAM10 and ADAM17 in the native cell membrane. Only for ADAM10, we find evidence for a physical interaction with APP, providing an explanation for the dominant role of ADAM10 in APP α-processing.

## Results

### Secretases are active in plasma membrane sheets

Nascent APP traffics through the constitutive secretory pathway to the plasma membrane, from which it is rapidly internalized, followed either by intracellular trafficking to the trans-Golgi network or back to the cell surface (4). In steady state, only a minor fraction of APP localizes to the plasma membrane (28, 29), where α-secretases are active.

**Figure 1:**
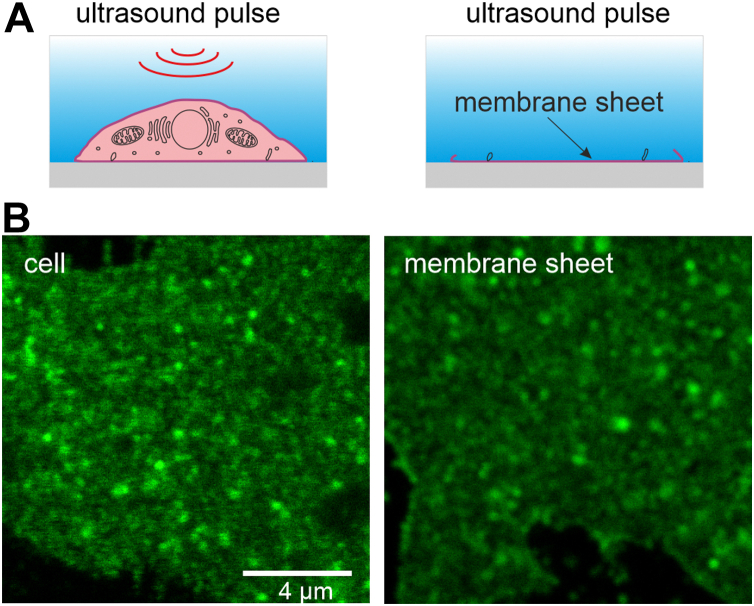
**Illustration of cell-freeplasmamembrane sheets** (30)**.***A*, membrane sheets are generated by a brief 100 ms ultrasound pulse in ice-cold solution. Mechanical shearing forces applied to glass-adhered cells (*left*) remove the apical membrane and cytosolic structures; only the basal plasma membrane remains (*right*). *B*, about 1 day after transfection, HepG2 cells expressing APP-GFP are either directly fixed (cell; *left*) or membrane sheets are generated (*right*), followed by recording of APP-GFP fluorescence by confocal microscopy. APP, amyloid precursor protein.

For characterizing the distribution of α-secretases and APP specifically at the cell surface, we employ microscopy on 'unroofed cells' (30), also referred to as plasma membrane sheets. They are generated by a brief ultrasound pulse applied to cells grown on glass coverslips, leaving behind a flat basal plasma membrane (see *cartoon* in Fig. 1*A*). Confocal microscopy is not required for optical sectioning, allowing for imaging at high signal-to-noise ratio. The method is used for decades studying plasma membrane architectures (30). More recently, they are used as well for studying APP clusters (29, 31) that are not an artifact of the preparation, as clusters are also observed in intact cells (31, 32) (Fig. 1*B*). However, to exclude that the ultrasound treatment may strongly affect secretases, we examined whether APP cleavage is still ongoing in membrane sheets.

**Figure 2:**
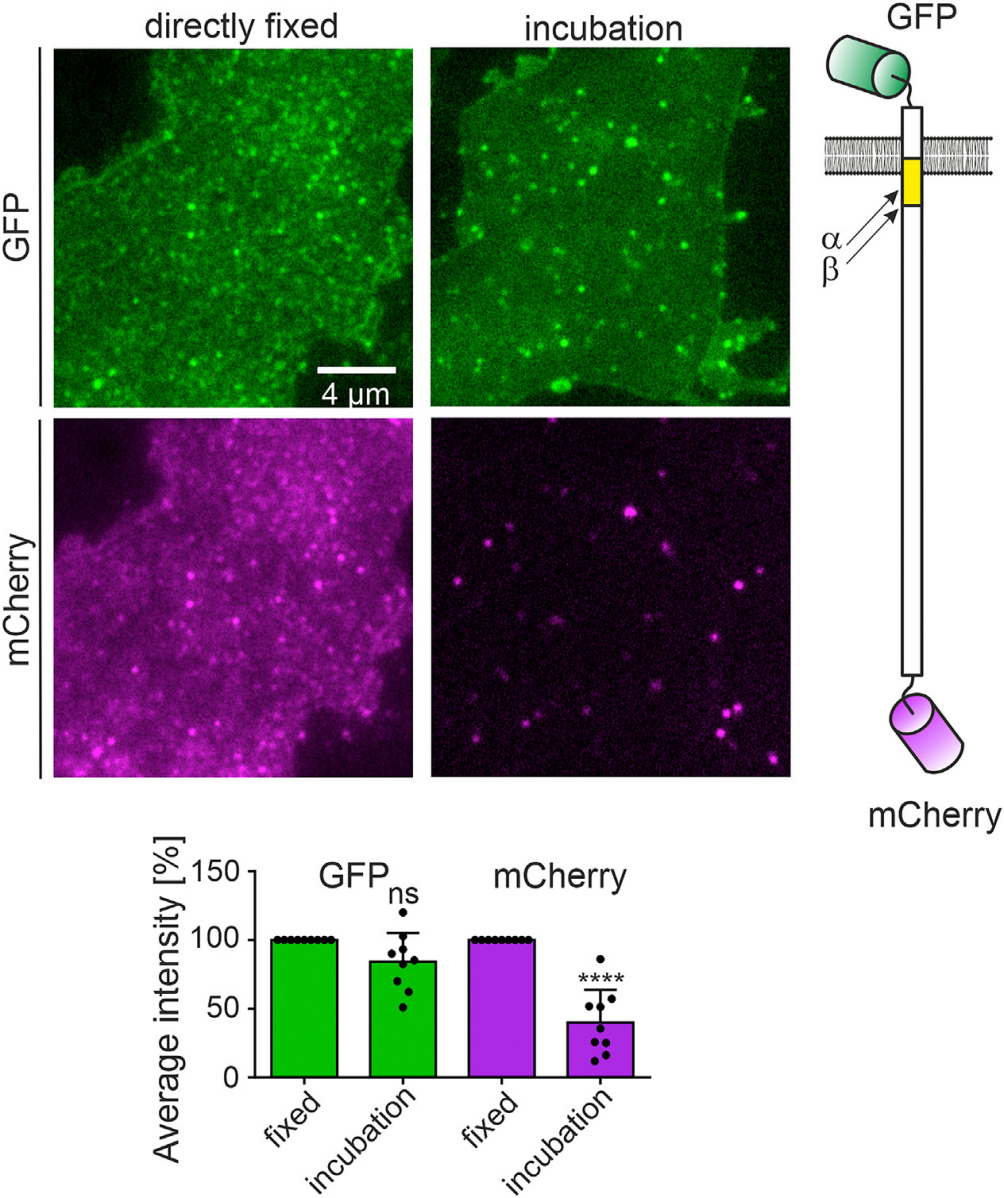
**APP processing in native plasma membrane sheets.** Epifluorescence micrographs show images from membrane sheets generated from HepG2 cells that express mCherry-APP-GFP (see illustration to the *right*; *arrows* point towards the α- and β-cleavage site; *yellow*, Aβ region). Membrane sheets are either directly fixed (*left images*) or after incubation for 10 min at 37 °C in medium containing 10 μM DAPT (*right images*). Images from the same channels are shown at the same contrast adjustment (*green*, GFP channel; *magenta*, mCherry channel). *Bar chart,* quantification of GFP and mCherry on directly fixed (set to 100%) or incubated membranes. Values are given as means ± SD (n = 9 experiments, 10–20 membrane sheets per experiment and condition). Unpaired Student’s *t* tests compare incubation to direct fixation (ns, *p* >0.05; ∗∗∗∗*p* <0.0001). Aβ, amyloid β-peptide; APP, amyloid precursor protein; DAPT, *N*-[*N*-(3,5-difluorophenacetyl)-l-alanyl]-S-phenylglycine *t*-butyl ester; ns, not significant.

To this end, HepG2 cells express the neuronal isoform APP_695_ that is double tagged with mCherry (monomeric cherry fluorescent protein) and green fluorescent protein (GFP; see illustration of the construct in Fig. 2). Membrane sheets are generated 21 h after transfection in ice-cold buffer, which immediately stops intracellular trafficking. Then, native membranes are either directly fixed or incubated for 10 min in a cell incubator in medium containing the γ-secretase inhibitor *N*-[*N*-(3,5-difluorophenacetyl)-L-alanyl]-S-phenylglycine *t*-butyl ester (DAPT). Under these conditions, we expect any occurrence of cleavage caused rather by α-secretases, as β-secretases are active at low pH (12, 33). In any case, cleavage yields a soluble mCherry-tagged ectodomain that is washed off, whereas the GFP-tagged CTF remains in the cell membrane. Hence, in comparison to directly fixed membranes, mCherry fluorescence diminishes upon processing, in contrast to GFP fluorescence. In our experiment, we observe a strong diminishment of the mCherry signal, whereas the GFP signal is unchanged (Fig. 2), showing that the functional interaction between APP and secretases persists in plasma membrane sheets.

For studying the association between APP and secretases in more detail, in the following, we use in addition to the non-neuronal HepG2 cell line the neuronal cell line SH-SY5Y as well.

### ADAM10 is the mainly active α-secretase

Several members of the ADAM family exhibit α-secretase activity; however, ADAM10 is mostly responsible for APP α-processing, albeit the extent of ADAM10 processing depends on the experimental system. To find out to which extent ADAM10 dominates α-processing in HepG2 and SH-SY5Y cells, we compared the broad inhibitor Batimastat (34) to the ADAM10-specific inhibitor GI254023X (35). In case all activity is based on ADAM10, both inhibitors would diminish to the same extent α-processing.

**Figure 3:**
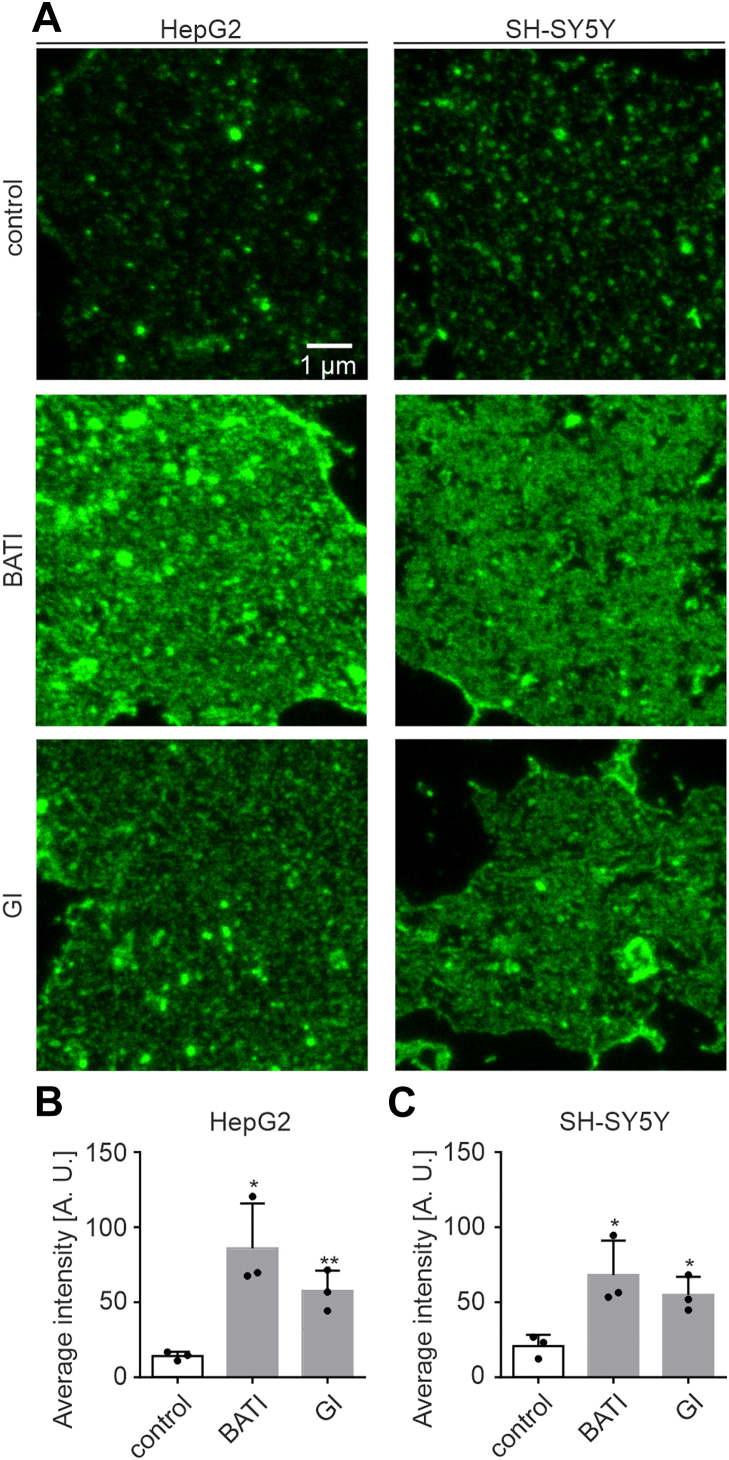
**ADAM10 is the mainly involved α-secretase in APP processing.***A*, STED micrographs of membrane sheets from HepG2 (*left*) and SH-SY5Y cells (*right*) expressing APP-GFP, incubated without inhibitor (control; *top*), the broad inhibitor Batimastat (BATI; *middle*), or the ADAM10-specific inhibitor GI254023X (GI; *bottom*). *B* and *C*, Atto647 nanobody intensity quantified on (*B*) HepG2 and (*C*) SH-SY5Y cell membrane sheets. Values are given as means ± SD (n = 3 experiments per cell line, 13–40 membrane sheets per experiment and condition). Unpaired Student’s *t* tests compare the control to BATI or GI (∗∗∗∗*p* <0.0001; ∗∗∗*p* <0.001; ∗∗*p* <0.01; and ∗*p* <0.05). ADAM, a disintegrin and metalloproteinase; APP, amyloid precursor protein; STED, stimulated emission depletion.

APP-GFP is overexpressed in HepG2 and SH-SY5Y cells, and the effect of the inhibitors on the APP level is quantified by stimulated emission depletion (STED) microscopy (Fig. 3*A*). Super-resolution microscopy is required for better resolving single APP clusters (Fig. S1), which is a prerequisite for analyzing the association between APP and secretases (see later). In both cell types, inhibitors increase strongly the average APP-GFP signal over control levels (Fig. 3). The effect of the specific ADAM10 inhibitor GI254023X is 60% of the Batimastat effect in HepG2 cells (Fig. 3*B*) and 70% in SH-SY5Y cells (Fig. 3*C*). This is in line with the expectation that also in our cellular systems ADAM10 is the main α-secretase responsible for APP processing. For the non-ADAM10 activity, several other α-secretases may be responsible, such as ADAM9, ADAM17, and ADAM19.

### Association between APP and α-secretases

Several explanations are conceivable for the dominant role of ADAM10 in APP processing. First, ADAM10 may be more abundant. Second, ADAM10 may locate closer to its substrate APP, or third, ADAM10 may be linked, either directly or indirectly, more tightly to APP.

For addressing these questions, we employed STED microscopy for studying the distribution of ADAM10 in comparison to ADAM17, an α-secretase that is involved in regulated APP processing (18) and constitutive α-cleavage when overexpressed (36). We analyzed the number and distribution of APP and ADAM10/17 maxima (for the technical definition of an intensity maximum, see Experimental procedures section). As previously shown (29), APP maxima represent crowds of APP molecules (referred to as APP clusters), which may not be the case for secretase maxima (for details, see Discussion section).

**Figure 4:**
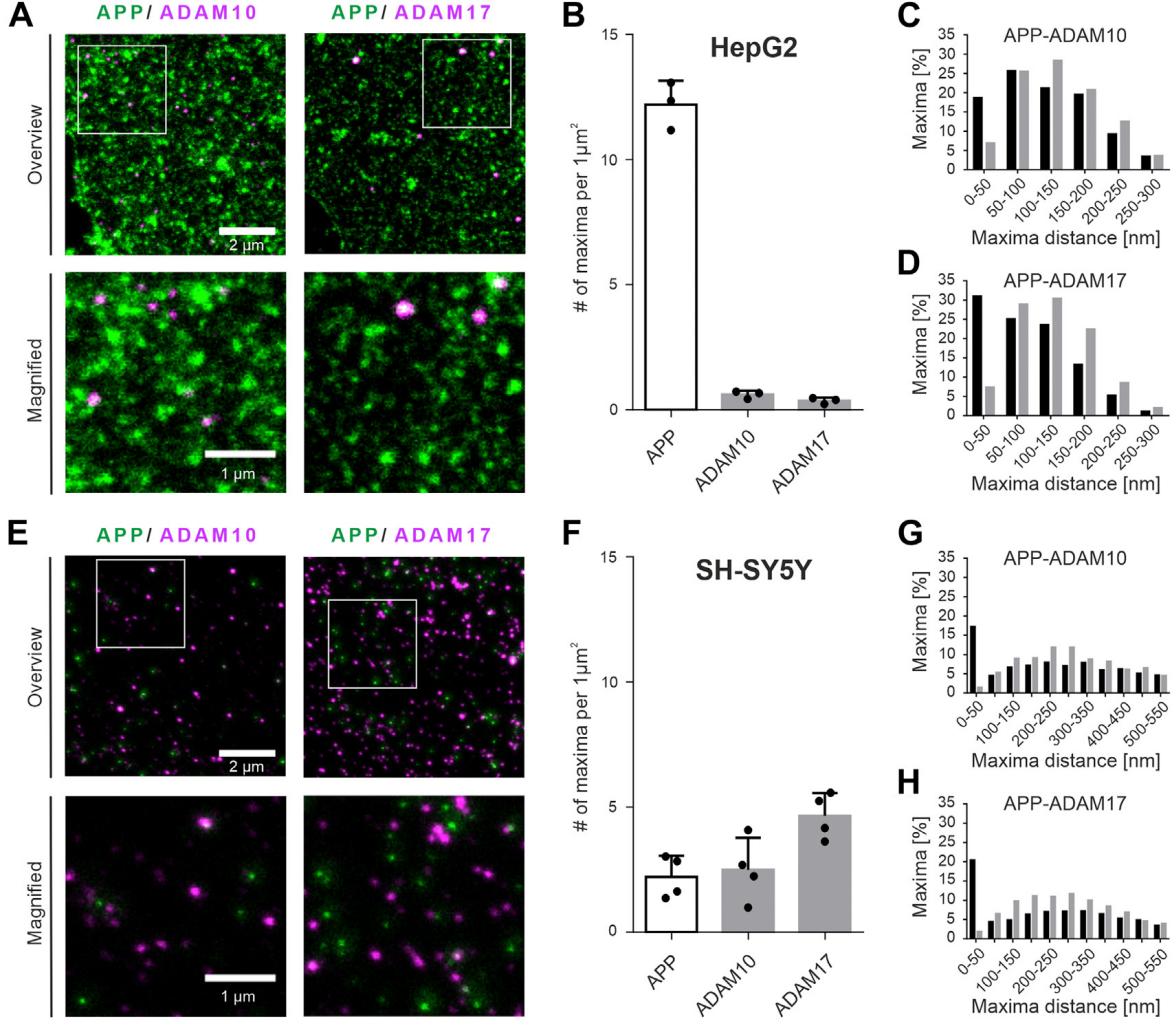
**Lateral organization of APP and secretases.** STED micrographs of membrane sheets from (*A*) HepG2 cells expressing APP-GFP and (*E*) nonoverexpressing SH-SY5Y cells. Membrane sheets are double stained for secretases (*magenta*; *left* ADAM10; *right* ADAM17) and (*A*) overexpressed or (*E*) endogenous APP (*green*). Shown are channel overlays of overviews (*top panels*) and magnified views from the boxed regions (*low panels*). *B* and *F*, APP and secretase maxima density. *C*, *D*, *G*, and *H*, frequency distribution histograms of shortest intermaxima distances between (*C* and *G*) ADAM10 and APP or (*D* and *H*) ADAM17 and APP maxima in HepG2 (*C* and *D*) and SH-SY5Y (*G* and *H*) cells. For clarity, only distances ≤300 nm (*C* and *D*) or ≤550 nm (*G* and *H*) are included. *Black* and *grey bars* show frequencies of original and flipped images (the images of one channel were flipped vertically and horizontally as reference for randomized distribution). *B* and *F*, values are given as means ± SD ([*B*] n = 3 experiments; [*F*] n = 4 experiments, 10–40 membrane sheets per experiment and condition). *C*, *D*, *G*, and *H*, histograms include data from 43 to 61 membrane sheets collected from three to four experiments. ADAM, a disintegrin and metalloproteinase; APP, amyloid precursor protein; STED, stimulated emission depletion.

Figure 4 shows the results obtained in HepG2 cells overexpressing APP-GFP and nonoverexpressing SH-SY5Y cells. The first impression is that in the two cell types the abundancy of APP and secretases is notably different in three aspects. First, in SH-SY5Y cells, APP and secretase maxima are present at densities in the same range, varying from 2.2 to 4.7 maxima per μm^2^ (Fig. 4, *E* and *F*). In contrast, in HepG2 cells, we find an up to ∼30-fold excess of APP over secretase maxima (Fig. 4, *A* and *B*), which is not only based on APP overexpression but as well on low secretase expression levels. The second difference is that secretase maxima are up to ∼13-fold more abundant in SH-SY5Y cells (compare Fig. 4, *B* and *F*), pointing toward strongly different expression levels in different cellular systems. Finally, in HepG2 cells, ADAM10 maxima are twice as frequent as ADAM17 maxima (Fig. 4*B*), whereas in SH-SY5Y cells, it is the other way around (Fig. 4*F*).

For evaluating the lateral association between APP and ADAM10/17 maxima, we determine the shortest distance between them. The maxima arise from clusters that have a certain physical size (*e.g.*, APP clusters have a diameter of ∼150 nm; Fig. S1*E*), and the resolution of the microscope is in the range of 60 to 90 nm (37). For these reasons, two intensity maxima arising from physically interacting APP and ADAM10 or ADAM17 entities (that may be either single molecules or larger ADAM-rich domains) are not expected to exhibit a zero distance to each other. Therefore, we consider ADAM maxima as being closely associated with APP maxima if their maxima distance is below 50 nm. In HepG2 cells, the fraction of closely associated secretase maxima is ∼19% (ADAM10) and ∼31% (ADAM17) (Fig. 4, *C* and *D*). Relating these percentages to the secretase maxima density (Fig. 4*B*), we obtain each 0.11 ADAM10 and ADAM17 maxima per μm^2^ that are potentially in physical contact with APP. In SH-SY5Y cells, we find closely associated ∼17% and ∼20% of the ADAM10 and ADAM17 maxima (Fig. 4, *G* and *H*), respectively. Relating these percentages to the total secretase maxima per μm^2^ (Fig. 4*F*), we obtain values of 0.43 ADAM10 and 0.95 ADAM17 maxima per μm^2^.

This analysis speaks against the idea that a closer association between ADAM10 and APP explains the dominant role of ADAM10 in α-processing, in particular in SH-SY5Y cells. Here, although 70% of processing is mediated by ADAM10 (Fig. 3*C*), the maxima distance analysis reveals that ADAM10 associates with APP actually less frequently. Therefore, we conclude that the lateral organization of APP and secretases provides no explanation why ADAM10 is the main secretase. The same accounts for the overall abundancy of secretases. In SH-SY5Y cells, ADAM17 maxima are twice as much abundant as ADAM10 maxima; still, 70% of APP is processed by ADAM10.

### A physical link between ADAM10 and APP

Next, we probed for a physical interaction between APP and the ADAMs. A classical method is coimmunoprecipitation. However, we were not able to coprecipitate ADAM10 with APP-GFP, and surprisingly, we also found no reports in the literature about any ADAM10–APP or ADAM17–APP coimmunoprecipitation. Such experiments may be unfeasible for several reasons. First, the ADAM–APP complex is an enzyme–substrate complex and presumably short lived because APP is cleaved shortly after binding. Second, even if the cleavage rate would be very slow or cleavage could be stopped, the complex may dissociate during the usually long-lasting coimmunoprecipitation. Third, a prerequisite for the protein–protein interaction may be an intact cell membrane that is dissolved during cell lysis.

**Figure 5:**
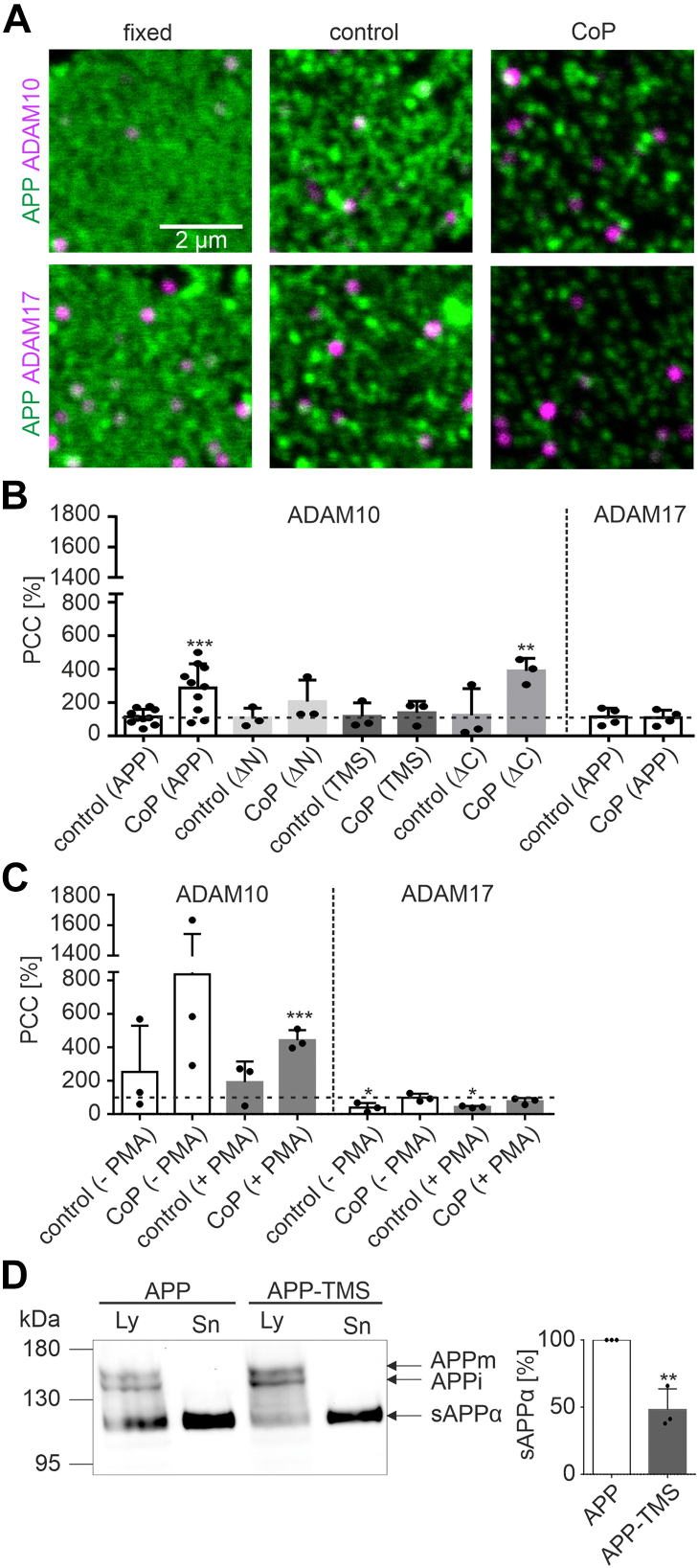
**APP coaggregation with ADAM10 and α-cleavage depend on the transmembrane segment of APP.***A*, confocal micrographs of membrane sheets from HepG2 cells expressing APP-GFP. Membrane sheets were directly fixed (*left*), incubated without (control; *middle*) or with cross-linking antibodies (CoP; *right*), followed by immunostaining for ADAM10 (*upper panels*) or ADAM17 (*lower panels*). APP-GFP (*green*) and ADAM10/ADAM17 (*magenta*). *B*, overlap through cross-linking is quantified by the Pearson correlation coefficient (PCC) between ADAM10 or ADAM17 and the APP constructs as indicated (APP, APP-ΔN, APP-TMS, and APP-ΔC; for membrane sheets from cells expressing the variants, see Fig. S7*A*). *C*, analysis as in (*B*) of membrane sheets from APP-GFP–expressing HepG2 cells grown in the absence (−PMA) or the presence of 1 μM PMA (+PMA) (for images of the membrane sheets, see Fig. S4*B*). *B* and *C*, values are expressed as percentage of the condition 'fixed' (100% reference line). Values are given as the means ± SD ([*B*] n = 3 to 10 experiments for ADAM10 and APP/APP variants; n = 4 experiments for ADAM17 and APP; [*C*] n = 3 experiments; 10–20 membrane sheets per experiment and condition). *D*, Western blot quantification of sAPPα in lysate (Ly) and supernatant (Sn) of HepG2 cells grown in the presence of 10 μM DAPT, expressing either GFP-labeled APP or APP-TMS. The sum of sAPPα band intensities (Ly + Sn) is related to the sum of the band intensities of immature (APPi) and mature APP (APPm); APP-TMS is related to APP (set to 100%). Value is given as the mean ± SD (n = 3 experiments). Unpaired Student’s *t* tests compare (*B* and *C*) control and CoP to fixed or (*D*) APP-TMS to APP (∗∗∗∗*p* < 0.0001; ∗∗∗*p* < 0.001; ∗∗*p* < 0.01; and ∗*p* < 0.05). ADAM, a disintegrin and metalloproteinase; APP, amyloid precursor protein; DAPT, *N*-[*N*-(3,5-difluorophenacetyl)-l-alanyl]-S-phenylglycine *t*-butyl ester; PMA, phorbol 12-myristate 13-acetate; sAPPα, soluble APPα; TMS, transmembrane segment.

In order to avoid cell solubilization and minimize the duration of the experiment, we probed the APP–ADAM interaction directly in native membranes by antibody-induced coaggregation. In this assay, one of the interaction partners (APP) is cross-linked by antibodies, yielding a distribution with less maxima, a more aggregated pattern (compare control to coprecipitated (CoP) in Fig. 5*A*). Then, we analyze whether ADAM10 or ADAM17 is dragged passively into these aggregates, or in other words, whether ADAMs coaggregate.

For the experiment, we used membrane sheets generated from Batimastat-treated HepG2 cells expressing APP-GFP to increase the APP expression level at the membrane. APP is cross-linked *via* its GFP tag at 37 °C by two short subsequent 15 min incubations. The first cross-linking step is mediated by a mouse monoclonal anti-GFP antibody that can cross-link only two APP-GFP molecules. The second cross-linking step is mediated by a polyclonal goat–antimouse antibody that further cross-links the APP-GFP–primary antibody complexes. Afterward, samples are fixed, the secretases are visualized by immunostaining, and the aggregation pattern is recorded by confocal fluorescence microscopy (Fig. 5*A*).

For evaluation whether there is a cross-linking effect, we also analyzed the APP-GFP distribution by calculating its relative standard deviation of the mean (rSDM), a parameter describing the degree of signal clustering that upon aggregation increases (38). Compared with directly fixed membrane sheets, the rSDM doubles in the absence of antibodies (Fig. S2) because of spontaneous protein aggregation (Fig. 5*A*, compare fixed and control) as previously observed for other proteins (38). However, when antibodies are present, the rSDM triples (Figs. S2 and 5*A*, compare control and CoP). In conclusion, the image analysis and visual impression suggest that the antibody treatment cross-links APP molecules into more defined aggregates.

Occasionally, a reduction of GFP intensity was observed in control or cross-linked samples, which was not caused by enhanced cleavage within the aggregates (α-secretase cleavage potentially followed by γ-secretase cleavage and wash off of the cleavage products) because GFP intensity diminishes also when Batimastat is present during cross-linking (Fig. S3). GFP-self-quenching is probably responsible for this effect, which can occur upon oligomerization of GFP-labeled proteins (39, 40).

Whether ADAMs coaggregate with APP is analyzed by calculating the Pearson correlation coefficient (PCC) between the APP and the ADAM10/ADAM17 channel. In the absence of cross-linking antibodies (control), the PCC between APP and the ADAMs is the same as in directly fixed samples (Fig. 5*B*, 100% defines the PCC in directly fixed membranes). Hence, spontaneous aggregation of APP is not associated with an increase in PCC. With antibodies (CoP), the PCC between ADAM10 and APP strongly increases, whereas between ADAM17 and APP it remains the same (Fig. 5*B*). This suggests that during cross-linking, because of physical association, ADAM10 is dragged together with APP into the more defined APP aggregates. Stimulation of secretase activity by phorbol 12-myristate 13-acetate (PMA) did increase the production of sAPPα (Fig. S4*A*), which however was not accompanied by an increase in the PCC between APP and ADAM17 (Fig. 5*C*). The lack of ADAM17 coaggregation indicates there is no strong interaction between ADAM17 and APP. Importantly, the detected physical link between APP and ADM10 is the first difference providing an explanation why ADAM10 is more effective in APP α-processing than ADAM17.

We next asked which segment of the APP molecule is required for coaggregation. To this end, we compare to wildtype APP two deletion constructs lacking either the extracellular fragment (APP-ΔN; lacking amino acids 22–626) or the intracellular fragment (APP-ΔC; lacking amino acids 649–695 (31)). Moreover, we include one construct, APP-transmembrane segment (APP-TMS), in which the TMS of APP (amino acids 627–647) is exchanged by the TMS of the epidermal growth factor receptor (EGFR; amino acids 646–668). To exclude that the exchange of the TMS alters the topology of the protein, we tested whether in C-terminally GFP-tagged APP-TMS, the Aβ region is at the extracellular site as well. To stop membrane trafficking, cells were incubated on ice. Extracellular Aβ epitopes were labeled by an antibody raised against amino acids 1 to 16 of the Aβ region, followed by labeling with a STAR RED-labeled secondary antibody. Then, membrane sheets were generated, fixed, permeabilized, and the GFP tag was visualized by an Atto594-labeled nanobody. While the first labeling detects only extracellular epitopes, the GFP labeling visualizes APP/APP-TMS independent from the topology. If APP-TMS would be wrongly inserted into the membrane, with an intracellularly oriented Aβ region, we would stain only GFP, and the ratio between STAR RED and GFP staining is zero. However, we find for APP and APP-TMS, the same ratios (Fig. S5), indicating correct topology of the APP-TMS mutant. Moreover, it should be noted that amino acids 621 to 624 bind to cholesterol, being part of a loop that interacts with the membrane (41, 42). Although APP-TMS still contains this surface-associated loop, exchange of the TMS by the slightly longer EGFR-TMS might change the affinity of the loop to the cell surface. This could alter the diffusion behavior and as a result affect the interaction with ADAM10. To test for a possible change in diffusion behavior, we compared the plasmalemmal mobility of APP-TMS to APP, employing fluorescence recovery after photobleaching. Fluorescence of the GFP tag was bleached in a squared region of interest (ROI) placed onto the basal plasma membrane, and the recovery of fluorescence was monitored over time (Fig. S6). In case APP-TMS is less tightly anchored to the membrane, its mobility should increase, which however, we do not observe (Fig. S6). Hence, regarding topology and membrane mobility, the data suggest that APP-TMS is indistinguishable from APP.

Comparing in the cross-linking assay APP to APP-TMS, APP-ΔC, and App-ΔN, already in the directly fixed sample APP-ΔN exhibited a markedly more punctuate distribution pattern (Fig. S7) and consequently a three-fold larger rSDM. This is because APP-ΔN less efficiently exits the endoplasmic reticulum, and on plasma membrane sheets, endoplasmic reticulum–plasma membrane contact sites remain and are visible as bright spots (43). Nevertheless, cross-linking increased the PCC of APP-ΔN and APP-ΔC, but not of APP-TMS (Fig. 5*B*; for images and the corresponding rSDMs, see Fig. S7). These results indicate that the TMS of APP is required for the physical link between ADAM10 and APP.

We next aimed for a more precise mapping within the TMS, exchanging only a few amino acids. The TMS of APP differs from the two amino acids longer EGFR-TMS at four positions, 628 to 630, 634 to 639, 641 to 644 (this section is different in length), and 646 (Fig. S8*A*). Based on these deviations, three constructs were generated, exchanging in the APP-TMS positions 628 to 630 (APP[↔628–630]), 634 to 639 (APP[↔634–639]), and 641 to 644/646 (APP[↔641–644/646]) by corresponding amino acids of the EGFR-TMS (Fig. S8*A*). In the cross-linking assay, like APP-TMS, variants APP(↔628–630) and APP(↔634–639) did not coaggregate, whereas APP(↔641–644/646) showed a trend toward an increase in PCC (Fig. S8*D*). However, none of the variants coaggregated significantly stronger than the 'fixed' condition. Hence, the interaction is not mediated by a single amino acid or a small region but rather stabilized by several regions distributed along the TMS. One explanation why the whole APP transmembrane domain is important could be that along the transmembrane domain several GxxxG/GxxxG-like motifs are present *via* which APP forms dimers (44) (see also Refs. (45, 46) for other proteins; Fig. S8*A*). With the exception of one motif present in the juxtamembrane region, the other three motifs are lost in APP-TMS, two are lost in APP(↔628–630), one in APP(↔634–639), and none in APP(↔641–644/646) (Fig. S8*A*). Hence, loss of the GxxxG/GxxxG-like motifs could perturb the dimeric state that may be a prerequisite for cross-linking. To test for a possible role of GxxxG/GxxxG-like motifs, each of the five glycines defining the motifs (depicted in bold in Fig. S8*A*) was substituted by a leucine. However, all mutants behaved essentially as APP (Fig. S8*G*) indicating that the association between APP and ADAM10 is not regulated *via* GxxxG/GxxxG-like motif–dependent dimerization.

To test whether the behavior in the cross-linking assay correlates with APP processing, we compared α-secretase processing of APP to APP-TMS and the variants. Please note that after TMS exchange, the γ-cleavage site is gone. For comparing α-processing in the absence of γ-cleavage to constructs with a γ-cleavage site, the γ-secretase inhibitor DAPT (47) is added after cell transfection. One day after transfection, we analyzed by Western blot (WB) the growth medium and cell lysate using the antibody detecting amino acids 1 to 16 of the Aβ region. This antibody recognizes full-length APP and sAPPα but not soluble APPβ (the β-secretase cleavage product). Relating sAPPα to APP, we find that α-cleavage of APP-TMS is reduced by more than 50% (Fig. 5*D*) and to a lower extent in the TMS variants (Fig. S8, *B* and *C*). In the GxxxG mutants, no effect on α-cleavage was observed (Fig. S8, *E* and *F*). Based on the correlation between the cross-linking and α-cleavage assay, we suggest that the physical link between ADAM10 and APP is functionally important for α-processing.

## Discussion

### ADAM10 is the major α-processing enzyme

Studying the impact of ADAM10 overexpression, knockdown, or mutation on the amount of cleavage products and plaque formation (15, 19, 22, 23, 24, 25), a large body of evidence shows that ADAM10 is the physiologically most important α-secretase, accounting for 79 to 90% of constitutive α-secretase cleavage activity (22, 23, 24). In HepG2 and SH-SY5Y cells used in this study, comparing a broad inhibitor to an ADAM10-specific inhibitor suggests that ADAM10 alone is responsible for 60 to 70% of α-secretase-based processing (Fig. 3, *B* and *C*).

A higher APP substrate specificity of ADAM10 is unlikely because different ADAMs, as for example, ADAM10 and ADAM17, exhibit rather broad substrate specificity, with overlapping protein substrates, such as APP (48). Alternatively, protease activity may be regulated by the abundancy of secretases in the plasma membrane. Evidence for this hypothesis comes from a study showing that tetraspanin 15 regulates the cell surface expression and α-secretase activity of ADAM10 (49). However, secretase abundancy alone is not necessarily the only factor (see later).

### A physical link between APP and ADAM10 provides an explanation for the dominant role of ADAM10 in α-processing

Investigating in the plasma membrane secretase abundancy and distribution, we find in HepG2 cells twice as many ADAM10 than ADAM17 maxima (Fig. 4*B*) but a similar frequency of ADAM10 and ADAM17 maxima closely associated with APP (0.11 maxima per μm^2^ each for ADAM10 and ADAM17; see above). In SH-SY5Y cells, compared with ADAM10, ADAM17 maxima are roughly twice as much abundant (Fig. 4*F*), like the frequency of ADAM17 maxima closely associated with APP (0.43 and 0.95 maxima per μm^2^ for ADAM10 and ADAM17, respectively; see above). In particular for SH-SY5Y cells, secretase overall abundancy and proximity to APP do not explain that ∼70% of processing is mediated by ADAM10. Taken together, the lateral organization of secretases and APP provides no explanation for APP being predominantly cleaved by ADAM10.

This leaves as last possibility that APP is linked more tightly to ADAM10 than to ADAM17. Employing in native membranes antibody-induced cross-linking of APP increases the signal overlap between APP and ADAM10 (Fig. 5, *B* and *C*). We propose that during APP cross-linking, the enzyme ADAM10 is dragged into the APP aggregates because of a physical link connecting it to its substrate. We cannot differentiate between a direct interaction or an indirect interaction but safely conclude that no such link is detected for ADAM17 (Fig. 5, *B* and *C*). This explains the predominant role of ADAM10 in α-secretase-based APP cleavage.

We can only speculate why, despite of a lacking physical link, in directly fixed membrane sheets ADAM10 and ADAM17 locate equally close to APP. The concept of multiprotease complexes may provide an answer. It has been reported that between α-/β-secretases and γ-secretases binary complexes form, one study even finds α-/β-/γ-secretase ternary complexes (50). It is possible that binary complexes form between the α-secretases ADAM10 and ADAM17 that could be recruited to APP clusters *via* an APP–ADAM10 interaction, explaining why both secretases are found in close proximity to APP.

The exact mechanism of processing yet is unclear. ADAM10 and ADAM17 have the same domain structure (Fig. S11) but share only ∼30% sequence identity (National Center for Biotechnology Information BLAST alignment tool, (51)); in the transmembrane domain, we find ∼55% conservation (Clustal Omega software with Gonnet PAM250 matrix (52, 53); Fig. S11). Although ADAM–substrate specificity does not rely on a specific amino acid signature (48) recognized by the catalytic protease domain, it is known that noncatalytic ADAM domains regulate substrate–enzyme interactions (54, 55) most likely because of steric hindrance. By this mechanism, the noncatalytic domains of ADAM10 and ADAM17 can affect the substrate-binding affinity and substrate cleavage site specificity (56). Stimulation of ADAM17 may cause a conformational change in the extracellular catalytic domain (57). However, stimulation with the phorbol ester PMA did not change the propensity of ADAM10 or ADAM17 to coaggregate with APP (Fig. 5*C*); therefore, we conclude that it does not regulate the physical interaction of the ADAMs with APP. Finally, the specificity may be defined by the different sequences of the secretase transmembrane domains (see aforementioned one; Fig. S11).

Likewise, the secondary structure of substrates can regulate the activity and substrate affinity of ADAM10 and ADAM17 (56). Therefore, the conformation of APP may also favor a higher substrate affinity of ADAM10 compared with ADAM17.

Finally, the physical link found between APP and ADAM10 could be indirect; other factors may bring substrate and secretase together. For example, tetraspanin 3 has been found to bind to ADAM10, APP, and the γ-secretase complex (58). Altogether, this points toward the possibility that tetraspanin 3 organizes tetraspanin-enriched microdomains (59) harboring, and by this physically linking together, APP and ADAM10.

### Role of APP dimerization

For the coaggregation of APP and ADAM10, the TMS of APP is essential (Fig. 5*B*), and the TMS is also required for processing of APP to sAPPα (Fig. 5*D*). Further narrowing down the relevant section of the TMS region failed because all tested three regions within the TMS are required for coaggregation and processing (Fig. S8, *A*–*D*). Another open question regarding the mechanism of the secretase–APP interaction is, whether APP dimerization is involved? For γ-cleavage, there is evidence that the dimer stability of the β-CTF (also referred to as C99) is important for interaction with and cleavage by the γ-secretase (60). Therefore, a dependence of the ADAM10–APP interaction on APP dimerization may be possible as well. However, single replacement of glycines in the dimerization mediating GxxxG/GxxxG-like motifs (45, 46) in the juxtamembrane and transmembrane regions of APP had no effect on the physical link of ADAM10 with APP and α-processing (Fig. S8, *E*–*G*). No effect on α-processing is in line with previous experiments showing that pairwise replacement of glycine to leucine in GG621/625LL, GG625/629LL, and GG629/633LL mutants had no effect on sAPPα levels as well (61).

### The molecular species involved in APP processing

As maxima of secretases were found to be closely associated with APP (Fig. 4), the question arises, what do the detected secretase maxima actually represent on the molecular level? Are they monomers, dimers or larger clusters? The ADAM maxima intensity is brighter as expected for single-molecule labeling, pointing to the presence of more than one molecule at the maxima location. Because the secretase maxima size is close to the resolution limit of the microscope (100–120 nm in SH-SY5Y cells and 110–150 nm in HepG2 cells), it is not possible to come up with a reliable estimate about the real physical size of the secretase maxima, clarifying whether maxima, for example, are large membrane domains, populated by many secretase molecules. Regarding APP maxima, it has been shown previously that in SH-SY5Y they contain 20 to 30 molecules concentrating on a 65 to 85 nm large spot (29) and could be structures forming in preparation for endocytosis (31). In any case, the secretase maxima likely reflect molecular species too large to enter the APP clusters, for which reason APP may be only accessible for processing at the periphery of the APP cluster.

### α-secretases as target for AD treatment

APP clustering might limit the accessibility of α-secretases to the APP substrate and therefore only allow for inefficient processing. Inefficient APP cleavage by α-secretases could motivate for a strategy using α-secretase processing in AD treatment. This approach is fundamentally different from β-secretase inhibition because it does not rely on inhibition but promotion of cleavage. By circumventing inhibition, biologically relevant non-APP sheddase products are not diminished, which could cause less side effects usually reported for β-secretase inhibitors (62). Another difference is that α-cleavage occurs at the cell surface, which is favorable as drugs must not enter the cell, like β-secretase inhibitors.

The idea of treating AD *via* regulating α-secretases has been suggested more than a decade ago (14, 15, 63). Understanding better the exact mechanism underlying APP α-processing opens the possibility to enhance the enzyme–substrate interaction at the cell membrane, boost α-processing, and by this shut down amyloidogenic processing. The finding that processing is mediated by the APP-TMS is a starting point in that direction.

## Conclusion

Here, we address the question why ADAM10 is the predominant secretase in APP α-processing. Using direct cross-linking of the substrate APP in the native plasma membrane, we find for ADAM10 a physical link to APP but not for ADAM17. We cannot differentiate in this assay if the physical link results from direct or indirect binding of ADAM10 to APP. For this purpose, additional experiments would be needed, for instance, showing in a minimal system that ADAM10 has a higher affinity to APP than ADAM17. However, such experiments would be difficult to perform as APP–secretase complexes are, as discussed previously, probably short-lived, and binding would be followed quickly by cleavage and disassembly of the complex. In any case, the cross-linking assay will allow for further exploration of the APP–secretase interaction, which could be important for establishing a new route for battling AD, using a strategy based on increasing α-secretase processing.

## Experimental procedures

### Antibodies

For immunostaining, primary rabbit polyclonal antibodies specific for ADAM10 (diluted 1:1000 in immunofluorescence [IF]; catalog no.: ab1997; Abcam) and ADAM17 (diluted 1:500 in IF; catalog no.: AB19027; Merck), a mouse monoclonal antibody raised against the CTF of APP (diluted 1:200 in IF, clone C1/6.1; catalog no.: 802801; BioLegend), and a mouse monoclonal antibody raised against β-amyloid amino acids 1 to 16 (diluted 1:100 in IF; clone 6E10; catalog no.: SIG-39320; Covance) were used. As secondary antibodies, we used donkey anti-rabbit coupled to AlexaFluor 594 (catalog no.: ab150064; Abcam), goat antimouse STAR RED (catalog no.: STRED-1001; Abberior Instruments), and goat anti-rabbit STAR RED (catalog no.: STRED-1002; Abberior Instruments), all diluted at 1:200. GFP was visualized by an Atto647N-labeled (catalog no.: gba647n-100; ChromoTek) or Atto594-labeled (catalog no.: gba594-100; ChromoTek) GFP-Booster, diluted at 1:200.

For cross-linking, a mouse monoclonal antibody raised against GFP (diluted 1:200; catalog no.: ab1218; Abcam) was used as primary antibody and IRDye 800CW coupled goat antimouse (diluted 1:500; catalog no.: 925-32210; Li-Cor) was used as secondary antibody.

For WBs, a primary mouse monoclonal antibody raised against β-amyloid amino acids 1 to 16 (see aforementioned one; diluted 1:2000 for WB; clone 6E10; catalog no.: SIG-39320; Covance) was used. IRDye 800CW goat antimouse (catalog no.: 925-32210; Li-Cor) diluted 1:10,000 served as secondary antibody.

### Plasmids

APP constructs are based on the sequence of human APP695 (National Center for Biotechnology Information reference sequence NM_201414). GFP-tagged APP (used in Figs. 1 and S1) and APP-ΔC (lacking amino acids 649–695; used in Figs. 5*B* and S7) are inserted into the pcDNA6.2 expression vector carrying the monomeric emerald GFP sequence and were described previously (31). pcDNA6.2-mCherry-APP-emGFP was previously described (64).

For pEGFP-C1-APP-mEGFP (used in Figs. 3, 4, *A*–*D*, 5 and S2–S10), APP was amplified using pcDNA6.2-APP-GFP as a template. The PCR product was inserted *via* the In-Fusion HD Cloning Kit (catalog no.: 638909; Takara) into a modified expression vector (65) consisting of pEGFP-C1 (catalog no.: 6076-1; Clontech Laboratories) with N-terminal monomeric-enhanced GFP (described previously (66)). From this plasmid, pEGFP-C1-APP-ΔN-mEGFP (lacking amino acids 22–626 of APP; used in Figs. 5*B* and S7) was produced by amplification of the whole pEGFP-C1-APP-mEGFP plasmid excluding amino acids 22 to 626 of the APP protein, followed by DpnI digestion of the template plasmid (DpnI, catalog no.: R0176S; New England Biolabs) and phosphorylation (T4 polynucleotide kinase, catalog no.: M0201S; NEB) and ligation (T4 DNA ligase, catalog no.: M0202S; NEB) of the PCR product. For pEGFP-C1-APP-TMS-mEGFP (used in Figs. 5 and S5–S10), the TMS of APP (amino acids 627–647) was exchanged by the TMS of the EGFR (NM_005228.5; amino acids 646–668). To this end, pEGFP-C1-APP-mEGFP was amplified excluding amino acids 627 to 647 of APP but with 15 nt overhangs encoding for amino acids of the EGFR-TMS. The EGFR-TMS was amplified by PCR, and both PCR products were merged using the In-Fusion HD Cloning Kit (catalog no.: 638909; Takara). The APP variants (used in Figs. S6–S7) APP(↔628–630) (I628A, G629T, L630G), APP(↔634–639) (G634A, V635L, V636L, I637L, A638L, T639L), APP(↔641–644_646) (I641V, V642A, I643L, T644G, V646F), APP-G621L, APP-G625L, APP-G629L, APP-G633L, and APP-G634L were created by mutagenesis PCR using pEGFP-C1-APP-mEGFP as template employing forward and reverse primers annealing back to back and introducing nucleotide substitutions in the transmembrane domain of APP. The PCR product was phosphorylated and ligated into pEGFP-C1-APP-mEGFP digested by DpnI.

### Cell culture and transfection

HepG2 cells (catalog no.: 300198; Cell Lines Service) were maintained and propagated essentially as described previously (67). SH-SY5Y cells were obtained from American Type Culture Collection at passage 26 (catalog no.: CRL-2266) and cultured as described previously (29)*.*

For transfection, cells were washed with Dulbecco's PBS (catalog no.: P04-36500; PAN-Biotech) followed by treatment with trypsin (catalog no.: P04-36500; PAN-Biotech) for about 2 min at 37 °C. Transfection was performed with the Neon-Transfection System (Thermo Fisher Scientific) using 100 μl gold tips containing 1.8 × 10^6^ cells per transfection mixed with 12.5 μg plasmid. A single pulse of 1200 V (for HepG2 cells) or 1100 V (for SH-SY5Y) with 50 ms width was applied. For microscopy, approximately 3 × 10^5^ cells were plated per 25 mm diameter glass coverslips coated with 100 μg/ml poly-l-lysine (catalog no.: P6282; Sigma–Aldrich). If indicated, 10 μM Batimastat (catalog no.: SML0041; Sigma–Aldrich), 3 μM GI254023X (catalog no.: SML0789; Sigma–Aldrich), 10 μM DAPT (catalog no.: D5942; Sigma–Aldrich), or 1 μM PMA (catalog no.: P1585; Sigma–Aldrich) dissolved in dimethyl sulfoxide were added 1 h after transfection to the growth medium, and dimethyl sulfoxide only was added to controls. For WB analysis, 4 × 10^6^ cells in 1.5 ml growth medium without antibiotics and serum were seeded per well in a 6-well plate (catalog no.: 83.3920.005; Sarstedt, Inc).

### Preparation of membrane sheets

Membrane sheets were generated 21 h after transfection or seeding. For membrane sheet generation, cover slips were washed twice with ice-cold Dulbecco's PBS (catalog no.: P04-36500; PAN-Biotech) and placed into a sonication chamber filled with ice-cold sonication buffer (120 mM potassium glutamate, 20 mM potassium acetate, 10 mM EGTA, 20 mM Hepes, pH 7.2). For sonication, we applied a 100 ms sonication pulse with 15% power (for SH-SY5Y cells) or 80% power (for HepG2 cells), essentially as described previously (29, 38).

### Immunostaining

For immunostaining, membrane sheets were fixed at room temperature (RT) for 30 min in 4% paraformaldehyde (PFA) in PBS, 137 mM NaCl, 2.7 mM KCl, 10 mM Na_2_HPO_4_, 1.76 mM KH_2_PO_4_, pH 7.4) followed by PFA quenching for 20 min with 50 mM NH_4_Cl in PBS. Then, membrane sheets were permeabilized with 0.2% Triton X-100 in PBS for 2 min, followed by blocking with 4% bovine serum albumin (BSA; catalog no.: P06-1391100) in PBS for 1 h at RT. Afterward, cover slips were incubated with primary antibody diluted in 1% BSA–PBS for 1 h at RT, followed by three washing steps with 0.5% BSA–PBS, and incubation with secondary antibody diluted in 1% BSA–PBS. Finally, samples were washed three times in PBS. For confocal and STED microscopy, for detection of the membranes from nonoverexpressing SH-SY5Y cells, Vybrant DiO Cell-Labeling Solution (catalog no.: V22886; Thermo Fisher Scientific) in a dilution of 1:200 in PBS or in overexpressing HepG2 cells, Rhodamine Phalloidin Reagent (catalog no.: ab235138; Abcam) in a dilution of 1:1000 was added, and cover slips were mounted on microscopy slides using ProLong Gold antifade mounting medium (catalog no.: P36930; Invitrogen).

### Assay of APP cleavage on unfixed membrane sheets

For evaluation of α-processing in plasma membrane sheets (Fig. 2), membrane sheets were generated from cells expressing mCh-APP-GFP 21 h after transfection. After sonication, coverslips were either directly fixed in 4% PFA–PBS or incubated in medium supplemented with the γ-secretase inhibitor DAPT (10 μM) for 10 min at 37 °C. After incubation of native membrane sheets, coverslips were fixed and quenched as described previously.

### Probing the membrane topology of APP and APP-TMS

Membrane sheets were generated from cells expressing GFP-labeled APP or APP-TMS 21 h after transfection. Extracellular epitopes were stained by incubation of living cells for 2 h at 4 °C employing a mouse monoclonal antibody raised against β-amyloid amino acids 1 to 16 (diluted 1:100 in 1% BSA–PBS; clone 6E10; catalog no.: SIG-39320; Covance). After three washing steps with 0.5% BSA–PBS at 4 °C, cells were incubated with goat antimouse STAR RED (diluted 1:200 in 1% BSA–PBS; catalog no.: STRED-1001; Abberior Instruments) for 2 h at 4 °C. Then, membrane sheets were generated, fixed, quenched, and permeabilized as described previously. After blocking with 4% BSA–PBS, membrane sheets were stained for GFP using an Atto594-labeled GFP-Booster (catalog no.: gba594-100; ChromoTek) for 1 h at RT. Samples were washed three times in PBS, and cover slips were mounted on microscopy slides using ProLong Gold antifade mounting medium (catalog no.: P36930; Invitrogen), followed by imaging employing confocal microscopy.

### Cross-linking of microdomains on unfixed membrane sheets

For antibody-induced cross-linking experiments (68), membrane sheets were generated from cells grown in the presence of 10 μm Batimastat. After sonication, coverslips were either fixed in 4% PFA–PBS (the 100% reference condition) or transferred into 0.5% BSA–PBS for a few minutes. Samples were incubated at 37 °C for 15 min with mouse monoclonal anti-GFP (catalog no.: ab1218; Abcam) diluted 1:200 in 1% BSA–PBS, followed by two washing steps in 0.5% BSA at RT, and incubation for 15 min at 37 °C with goat–antimouse-IRDye800CW (catalog no.: 925-32210; Li-Cor) diluted 1:500 in 1% BSA–PBS (in the presence of Batimastat when indicated). In controls, antibodies were omitted. In total, the incubation time of unfixed membrane sheets was 45 min, before they were fixed in 4% PFA–PBS.

Then, the 100% reference, control, and coprecipitation samples were quenched and immunostained as described previously, using as primary antibody rabbit–anti-ADAM10 (catalog no.: ab1997; Abcam) or rabbit–anti-ADAM17 (catalog no.: AB19027; Merck) and as secondary antibody goat–anti-rabbit STAR RED (catalog no.: STRED-1002; Abberior Instruments).

### Fluorescence recovery after photobleaching analysis

HepG2 cells were transfected to express GFP-labeled APP or APP-TMS (see previous one) and grown for 21 h in the presence of 10 μM Batimastat. For imaging, cells were transferred to Ringer solution (130 mM NaCl, 4 mM KCl, 1 mM CaCl_2_, MgCl_2_, 48 mM d-glucose, 10 mM Hepes, pH 7.4) containing 10 μM Batimastat, where they remained for a maximum period of 40 min. Imaging was performed using an Olympus Fluo View 100 laser scanning microscope, essentially as described previously (38). In brief, the pixel size was set to 204 nm; image size was 100 × 100 pixel. An ROI with a size of 15 × 15 pixels was defined in the basal plasma membrane and bleached for a duration of 1.5 s using at the same time a 488 nm and 405 nm laser at their maximum intensity. In total, a sequence of 120 images was recorded at 1.77 Hz, containing three prebleach and 117 postbleach images. The average image intensity of the ROI was background corrected and normalized to the average of the prebleach values. Postbleach values were plotted against time, yielding recovery traces. Measurements exhibiting a vertical drift of the focal plane, identified by an intensity variation of >15% in a nonbleached reference ROI, were excluded from the analysis. All recovery traces from one biological replicate were averaged, and from the averaged recovery traces, the half-times of recovery (*t*_1/2_) were determined by fitting of a hyperbolic curve: *y*(*t*) = offset + maximal recovery *× t*/(*t* + *t*_1/2_).

### Epifluorescence microscopy

Fixed membrane sheets were imaged in PBS containing 1-(4-tri-methyl-ammonium-phenyl)-6-phenyl-1,3,5-hexatriene *p*-toluene-sulfonate (TMA-DPH; catalog no.: T-204; Invitrogen). Epifluorescence microscopy was performed using a Zeiss Axio Observer D1 epifluorescence microscope equipped with a Plan-Apochromat 100×/numerical aperture 1.4 oil immersion objective, and a 12 bit CCD camera (Sensicam QE; PCO AG) was used, yielding a pixel size of 64.5 nm. For illumination, a 75 W xenon arc lamp (N XBO 75; Zeiss) was employed using the filter sets F36-500 DAPI HC for TMA-DPH, F36-525 EGFP HC for GFP, and F36-503 TRITC HC (AHF Analysetechnik) for mCherry. Exposure times were 1000 ms for all channels.

### Confocal and STED microscopy

For confocal and STED microscopy, coverslips mounted on microscopy slides were imaged with a four-channel easy3D super-resolution STED optics module (Abberior Instruments) combined with an Olympus IX83 confocal microscope (Olympus) using an UPlanSApo 100× (1.4 numerical aperture) objective (Olympus). GFP and Vybrant DiO were excited with a 485 nm laser and recorded with combined 500 to 520 nm and 532 to 558 nm filters. Alexa594 and Rhodamine Phalloidin were excited with a 561 nm laser and recorded with a 580 to 630 nm filter. Atto647N and STAR RED were excited with a 640 nm laser and detected with a 650 to 720 nm filter. For STED microscopy, a pulsed 775 nm STED laser was used for depletion of Alexa594, STAR RED, and Atto647N.

A pinhole size of 60 μm was used, and the pixel size was 25 nm for all images. Confocal images were recorded with time-gated detection with 0.78 ns delay and 8 ns gate width. STED micrographs were recorded with six line accumulations and time-gated detection with 0.75 ns delay and 8 ns gate width.

### SDS-PAGE and WB

About 21 h after transfection, the 1.5 ml growth medium per well was centrifuged at 1000*g* for 3 min at 4 °C. Then, 0.5 ml of 4× sample buffer (277.8 mM Tris–HCl, 44.4% v/v glycerol, 4.4% v/v SDS, 0.02% w/v bromphenol blue, 5% v/v β-mercaptoethanol, pH 6.8) was added. The cells in the 6-well plate (catalog no.: 83.3920.005; Sarstedt) were scraped off with a cell scraper, washed two times with PBS by cell pelleting at 1000*g* for 3 min at 4 °C, and the cell pellet was resuspended in 80 μl 1× sample buffer. Samples were agitated at 95 °C for 10 min and then subjected to SDS-PAGE using a 4% polyacrylamide stacking gel and 8% running gel and blotted to a nitrocellulose membrane (catalog no.: HP40.1; Carl Roth; 0.2 μm pore size). Membranes were washed in PBS and blocked for 1 h with Intercept blocking buffer (catalog no.: 927-70001; Li-Cor). Then, membranes were incubated with primary antibodies diluted in Intercept blocking buffer supplemented with 0.1% v/v Tween-20 overnight at 4 °C. After washing four times with PBS supplemented with 0.1% Tween-20, membranes were incubated with secondary antibodies diluted in Intercept blocking buffer containing 0.1% Tween-20 for 1 h at RT. Thereafter, membranes were washed three times with PBS supplemented with 0.1% Tween-20 and one time with PBS. Bands were detected using the 700 and 800 nm channels of a Li-Cor Odyssey Classic Imaging System.

### Image analysis

Micrographs and WB images were analyzed with the ImageJ software (Wayne Rasband, National Institutes of Health).

Membrane sheets were selected in the respective channel. In case of two channels, membrane sheets were selected in the GFP and Vybrant DiO channel. For illustration of the fluorescence intensities, a linear lookup table was employed, using the same minimum and maximum value settings in all images from one experiment and one channel (*e.g.*, GFP). For calculating the average fluorescence intensity, rectangular ROIs were placed onto membrane sheets selected, calculating the mean intensity within the ROIs, and correcting for background values from ROIs placed next to the membrane sheet.

For analyzing the distribution of APP and secretases, we characterized their intensity maxima (maxima density, size, shortest distance, and intensity). To this end, a custom ImageJ macro was used, which is based on the “Find Maxima” ImageJ function that creates a binary mask using a threshold >1 to 5 a.u. pixel intensity (depending on the experiment and channel). Then, within the objects defined by the binary mask, the brightest pixels are identified (in the following referred to as “pixel maxima”; the position of the maxima is given in pixel coordinates). Before thresholding, to improve maxima detection, pixel noise was reduced by smoothing the images employing the function “Gaussian blur” (σ = 1).

Maxima intensities are determined by placing a five pixel circular ROI onto the “pixel maxima” and calculating the average fluorescence intensity in the ROI. The maxima positions are determined by calculating the center of mass of fluorescence within the ROIs, yielding subpixel resolution of the maxima positions. The shortest intermaxima distance was determined from the maxima positions. To this end, a custom-written ImageJ macro calculates based on the mathematical theorem of pythagoras for each maximum the distances to all other maxima and selects the shortest distance, as described previously (37). The maximum size is expressed as the full width at half maximum of a Gaussian fit, determined by line scan analysis. To this end, a vertical and a horizontal 15 × 3 pixel line scan was centered on the pixel maximum. Each intensity distribution was fitted to a Gaussian function. From the line scan featuring the best fit quality, the full width at half maximum was taken as maximum size. Maxima were excluded when the fit quality of *R*^2^ was lower than 0.8 and the peak was noncentered (not in the middle third of the line scan). For each individual membrane sheet, maxima values were averaged. The maxima density was determined by normalizing the number of maxima to the size of the analyzed ROI area.

The PCC between two channels was calculated from ROIs placed in one reference channel and propagated to the other respective channel using a custom ImageJ macro.

To obtain the rSDM, the standard deviation of the mean pixel intensity in an ROI was determined by ImageJ and related to the average fluorescence intensity.

For WB analysis, only bands of interest are displayed in Figures. 5*D*, S4*A*, and S8*B* (for full-length images of WBs, refer to Fig. S12). Freehand ROIs were drawn around the bands of interest, and band intensity was determined by measurement of the integrated fluorescence intensity within this ROI. The same ROI was moved to a region within the same lane with no visible band, and the measured integrated background intensity was subtracted from the respective band intensity. In the cleavage assay, the integrated fluorescence intensity of sAPPα was divided by the integrated fluorescence intensity of full-length APP (sum of mature and immature APP intensity), and wildtype APP was set to 100%.

## Data availability

All data generated or analyzed during this study are included in the article and supporting information.

## Supporting information

This article contains supporting information (31, 52, 53).

## Conflict of interest

The authors declare that they have no conflicts of interest with the contents of this article.
