# Supplementary material for: The transmembrane domain of the amyloid precursor protein is required for antiamyloidogenic processing by α-secretase ADAM10
Source: J Biol Chem. 2022 Apr 7;298(6):101911. doi: 10.1016/j.jbc.2022.101911 (PMC9127328; doi:10.1016/j.jbc.2022.101911)
Supplement: Supplemental Figures S1–S12 [file mmc1.docx]

**Supporting Information**

**The transmembrane domain of the amyloid precursor protein is required for anti-amyloidogenic processing by α-secretase ADAM10**

Lisa Hitschler^1^, Thorsten Lang^1*^

^1^Department of Membrane Biochemistry, Life & Medical Sciences (LIMES) Institute, University of Bonn, Carl-Troll-Straße 31, 53115 Bonn, Germany

^*^Correspondence should be addressed to thorsten.lang@uni-bonn.de


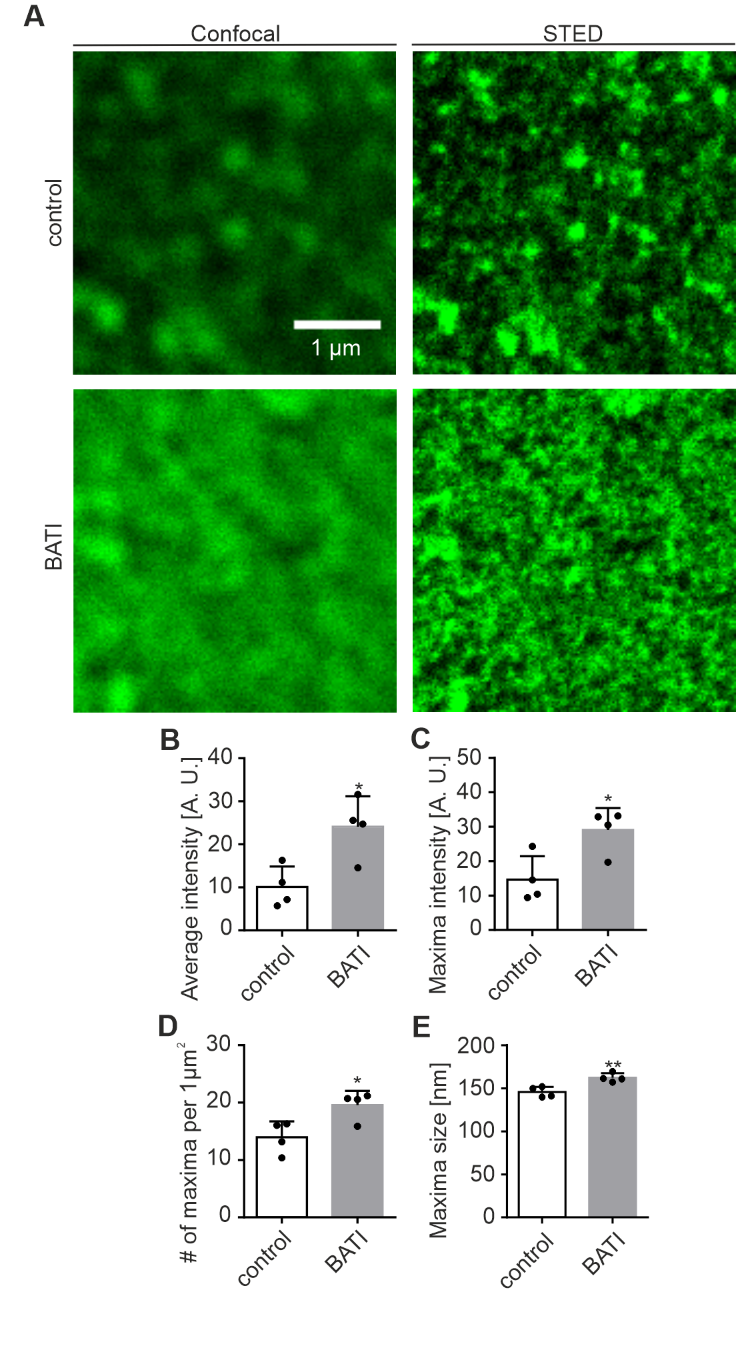


**Figure S1** *STED microscopy is required for properly resolving the APP cluster pattern.*

(A) Confocal (*left*) and STED (*right*) micrographs of membrane sheets from control (*top*) and 10 µM Batimastat treated (BATI; *bottom*) HepG2 cells expressing APP-GFP. We included the Batimastat condition to test whether an increase in the APP level affects APP clusters. APP-GFP is labelled for STED microscopy with an Atto647N-labelled nanobody raised against GFP. On STED micrographs, we quantified (B) the average Atto647N-intensity and the maxima (C) intensity, (D) density and (E) size. Batimastat increases maxima intensity, has a moderate effect on cluster density, and hardly any effect on maxima size. This is in line with a model that APP clusters are endocytic structures (32) with a size likely defined by the endocytic machinery. Therefore, an increase in APP molecules rather increases the molecule copy number per cluster, and to some extend the cluster number, instead of cluster size. Values are given as means ± SD (n = 4 experiments; 14 - 37 membrane sheets per experiment and condition). Unpaired Student’s t-tests compare control to BATI (****p <0.0001; ***p <0.001; **p <0.01; *p <0.05).


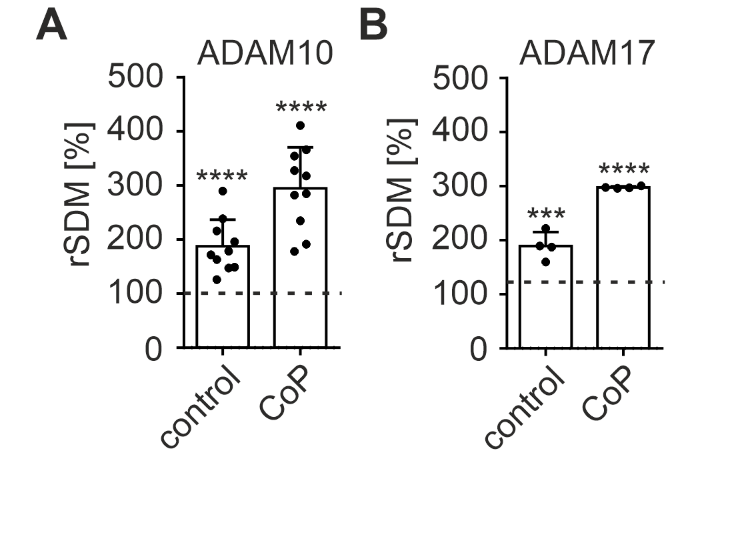


**Figure S2** *Cross-linking of APP-GFP causes a higher degree of signal clustering.*

Additional analysis of data shown in **Fig. 5**, evaluating the antibody-induced cross-linking effect. (A and B) Relative standard deviation of the mean (rSDM) of APP-GFP analyzing co-aggregation of (A) ADAM10 or (B) ADAM17. Values are related to the condition ‘fixed’ which defines the 100 % reference line. Values are given as means ± SD (A, n = 10; B, n = 4 experiments; 10 - 20 membrane sheets per experiment and condition). Unpaired Student’s t-tests compare control and CoP to fixed (****p <0.0001; ***p <0.001; **p <0.01; *p <0.05).


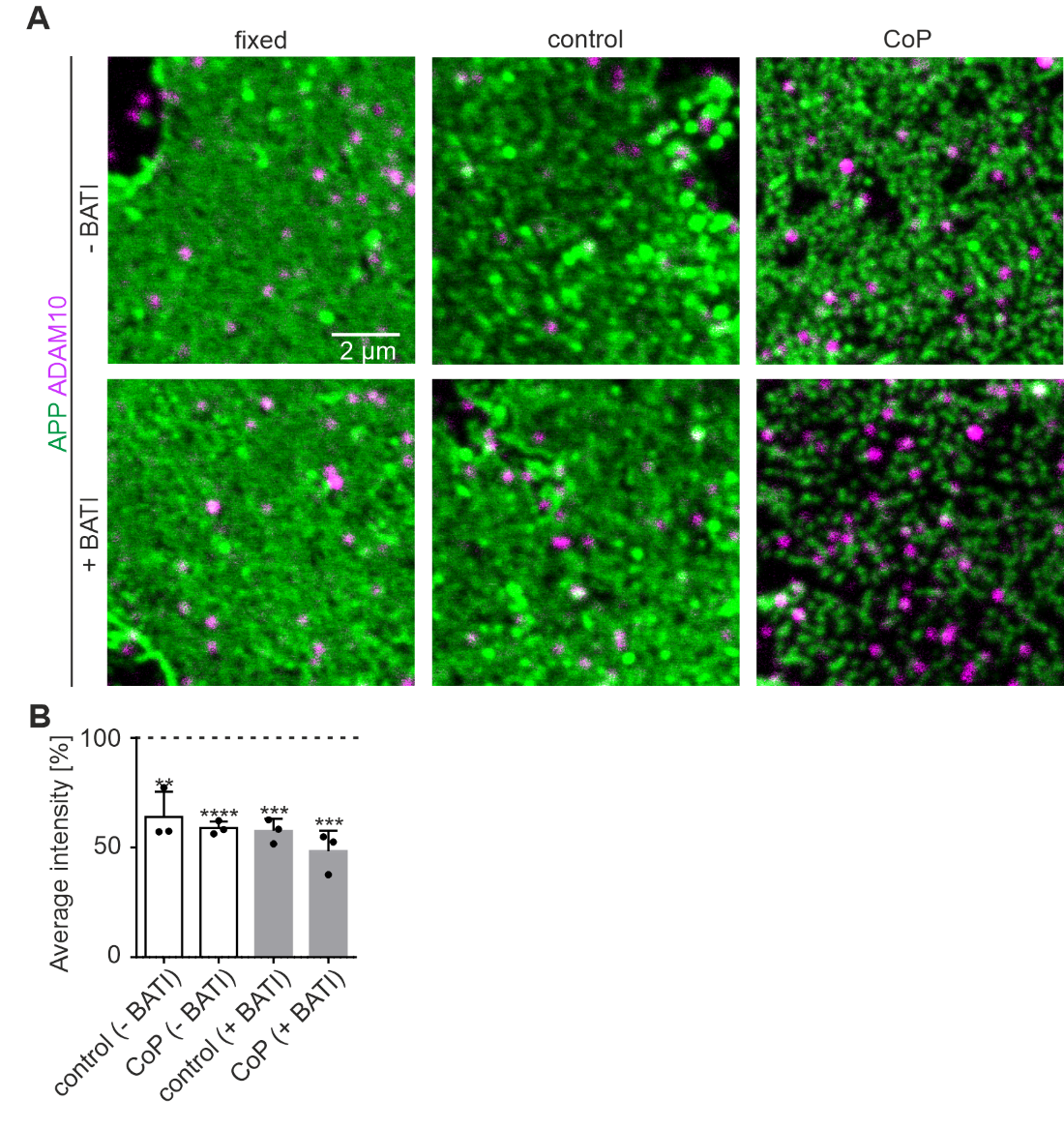


**Figure S3** *Antibody-induced cross-linking of APP in the presence of Batimastat.*

(a) Confocal micrographs of membrane sheets from HepG2 cells expressing APP-GFP directly fixed (*left panel*), or incubated without (control; *middle panel*) or with cross-linking antibodies (CoP; *right panel*) in the absence (- BATI) or presence of α-secretase inhibitor (+ BATI), followed by immunostaining for ADAM10. Images are overlays of the green (APP-GFP) and magenta (ADAM10) channel. (b) Average intensity of the APP-GFP intensity. The condition ‘fixed’ is set to 100% and displayed as reference line. Values are given as means ± SD (n = 3 experiments, 20 membrane sheets per experiment and condition). Unpaired Student’s t-tests compare fixed to control and CoP (****p <0.0001; ***p <0.001; **p <0.01; *p <0.05).


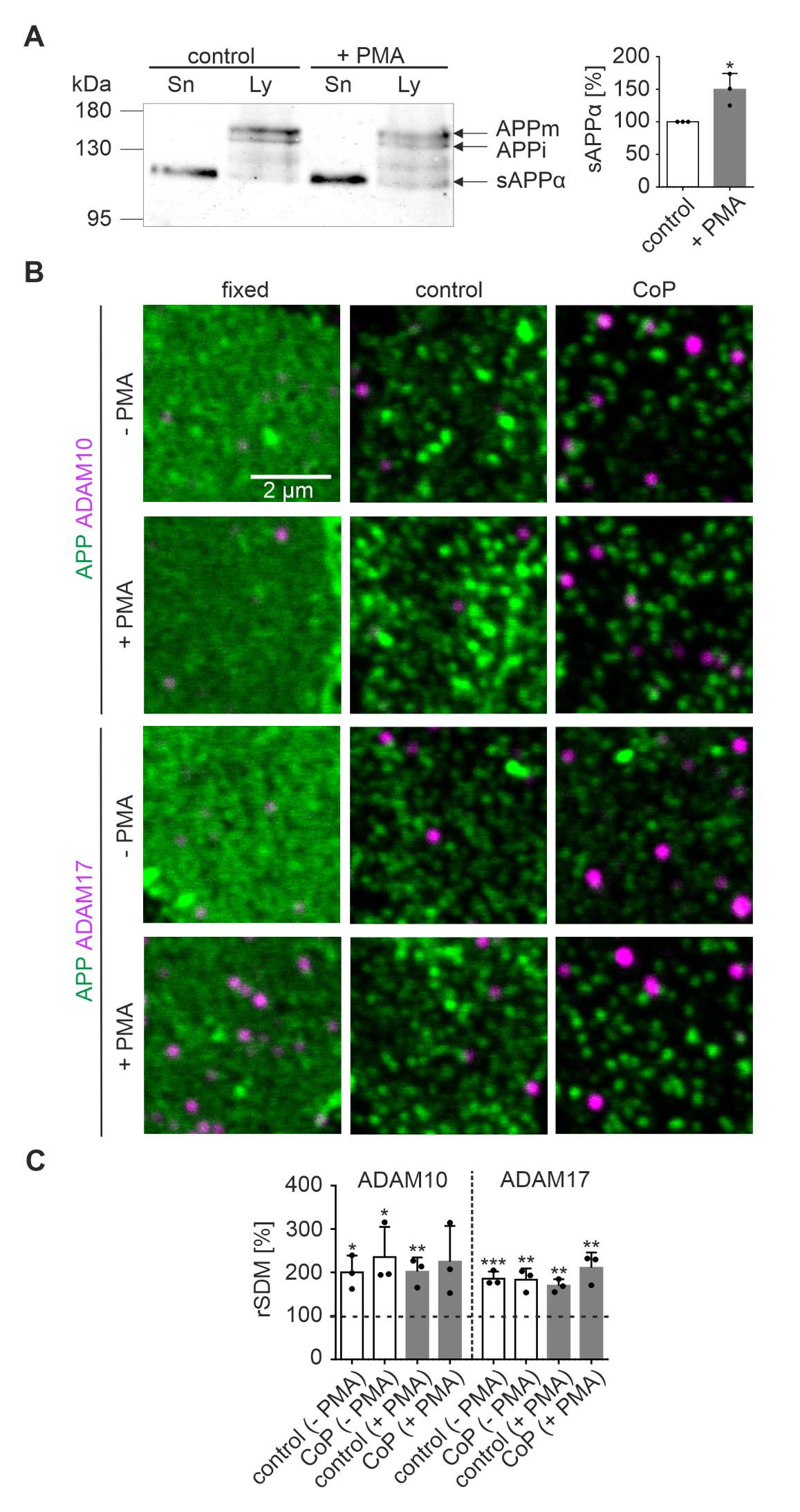


**Figure S4** *Phorbol ester stimulation of α-cleavage has no impact on physical interaction of APP with ADAM10 and ADAM17.*

(A) Western blot quantification of sAPPα in lysate (Ly) and supernatant (Sn) of HepG2 cells grown in the absence (control) or presence of 1 µM PMA (+ PMA), expressing APP-GFP. The sum of sAPPα band intensities (Ly + Sn) is related to the sum of the band intensities of immature (APPi) and mature APP (APPm); + PMA is related to control (set to 100%). Value is given as the mean ± SD (n = 3 experiments). Unpaired Student’s t-test compares + PMA to control (****p <0.0001; ***p <0.001; **p <0.01; *p <0.05). (B) Confocal micrographs of membrane sheets from HepG2 cells grown in the absence (- PMA) or presence of 1 µM PMA (+ PMA), expressing APP-GFP. Membrane sheets were directly fixed (*left*), incubated without (control; *middle*) or with cross-linking antibodies (CoP; *right*), followed by immunostaining for ADAM10 (*upper panels*) or ADAM17 (*lower panels*). APP-GFP (green), ADAM10/ADAM17 (magenta). (C) Relative standard deviation of the mean (rSDM) of APP. Values are expressed as percentage of the condition ‘fixed’ (100% reference line). Values are given as the means ± SD (n = 3 experiments; 15 - 20 membrane sheets per experiment and condition). Unpaired Student’s t-tests compare fixed to control and CoP (****p <0.0001; ***p <0.001; **p <0.01; *p <0.05). For PCC values see **Fig. 5C**.


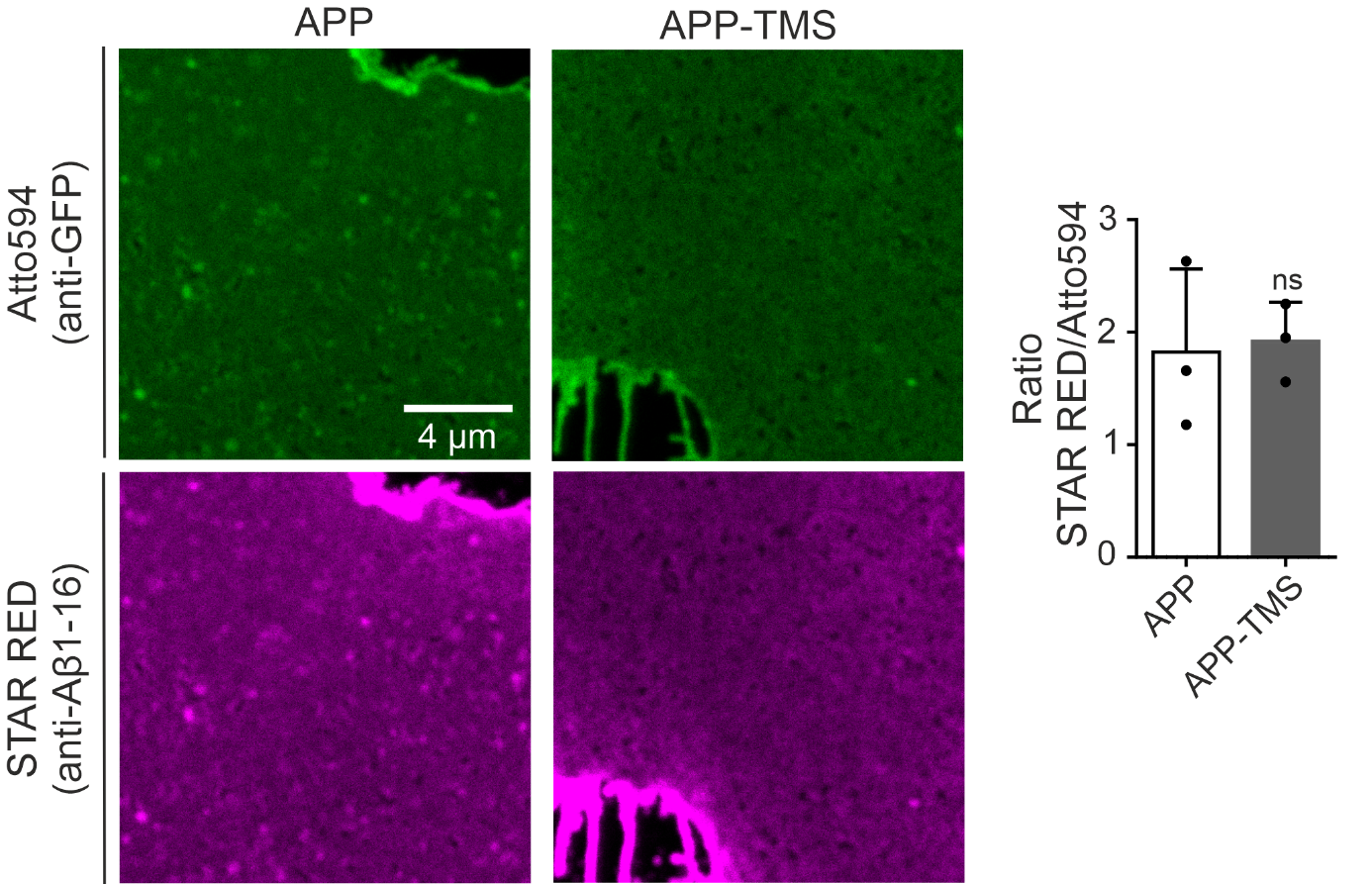


**Figure S5** *APP and APP-TMS have the same topology.*

HepG2 cells were transfected to express C-terminally labeled APP or APP-TMS. 21 h after transfection, cells were incubated at 4 °C with a primary antibody raised against an extracellular APP epitope (Aβ1-16), followed by incubation with a STAR RED labelled secondary antibody (magenta). In this step, APP-TMS is only stained if inserted correctly. Afterwards, membrane sheets were generated, fixed, permeabilized and stained for GFP (using an Atto594 labelled nanobody; green), followed by imaging. Confocal micrographs from the same channels are displayed at the same contrast adjustment. Bar chart; ratio of the average STAR RED and Atto594 intensity. Values are given as means ± SD (n = 3 experiments, 20 membrane sheets per experiment and condition). Unpaired Student’s t-tests compare APP-TMS to APP (****p <0.0001; ***p <0.001; **p <0.01; *p <0.05; ns (not significant) p >0.05).


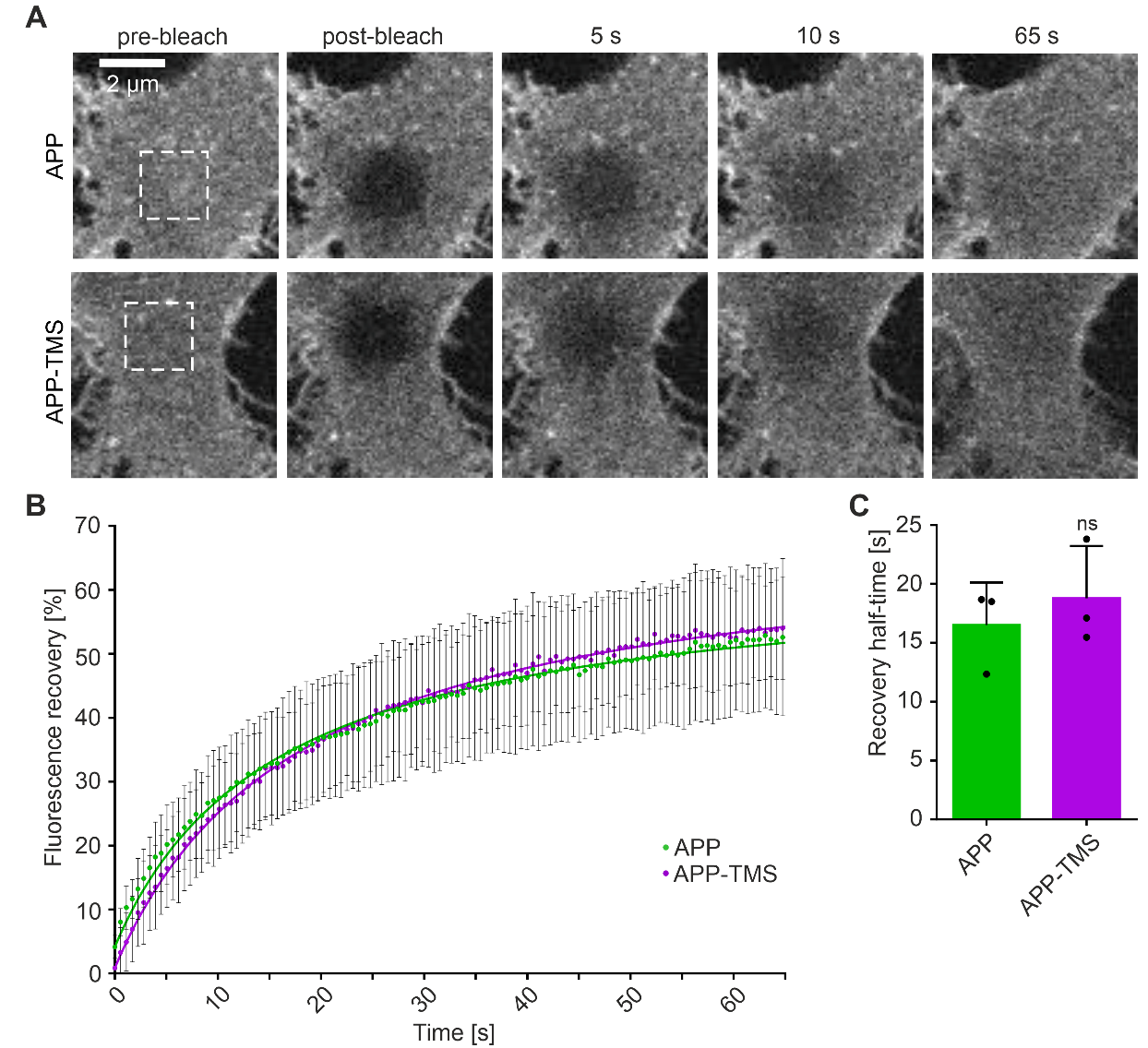


**Figure S6** *Plasmalemmal mobility of APP-TMS compared to wild-type.*

Confocal micrographs illustrating representative FRAP experiments from HepG2 cells transfected to express GFP-tagged APP (upper panels) or APP-TMS (lower panels), grown for 21 h in the presence of 10 µM Batimastat. Fluorescence bleaching was performed in a squared ROI (dashed square) at the basal plasma membrane. Shown are the frames preceding bleaching (pre-bleach; first column), directly after bleaching (post-bleach; second column) and 5 s (third column), 10 s (fourth column) and 65 s (fifth column) after bleaching. (B) The recovering GFP fluorescence of APP (green) and APP-TMS (magenta) is related to the pre-bleach value and plotted over time. (C) From the averaged normalized recovery traces of each experimental day the recovery half-time was calculated. (B and C) Values are given as means ± SD (n = 3 experiments, 15 cells per experiment and condition). (C) Unpaired Student’s t-tests compare APP-TMS to APP (ns (not significant) p >0.05).


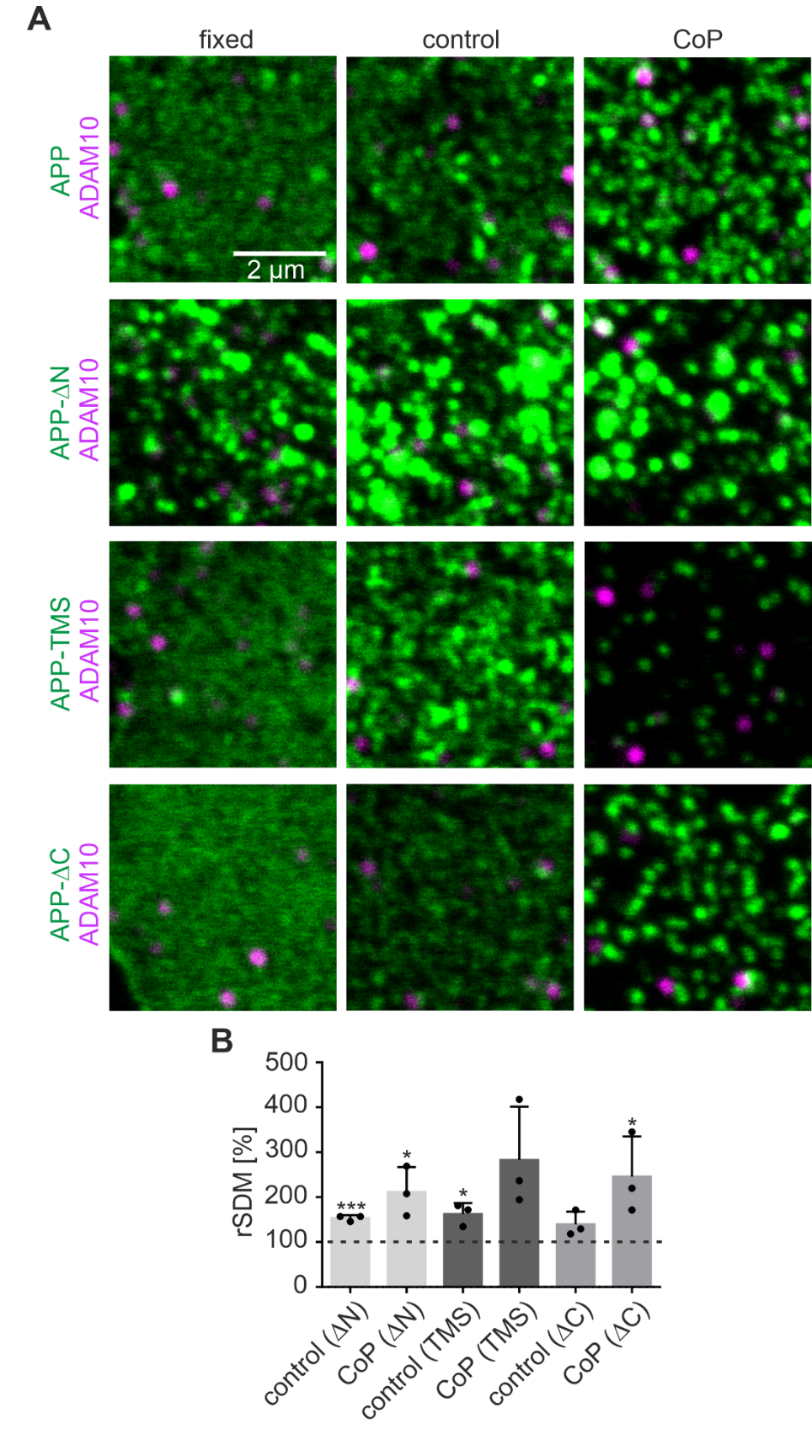


**Figure S7** *Cross-linking of APP-∆C, APP-∆N and APP-TMS*.

(A) Confocal micrographs of constructs not shown in **Fig. 5A** but analyzed in **Fig. 5B**. For comparison, membrane sheets of cells expressing APP-GFP are shown again. (B) Relative standard deviation of the mean (rSDM) of APP-∆N, APP-TMS and APP-∆C (values of APP are shown in **Fig. S2**). Values are related to the condition ‘fixed’ which is set to 100 % and displayed as reference line. Values are given as means ± SD (n = 3 experiments, 10 - 20 membrane sheets per experiment and condition). Unpaired Student’s t-tests compare fixed to control and CoP (****p <0.0001; ***p <0.001; **p <0.01; *p <0.05).


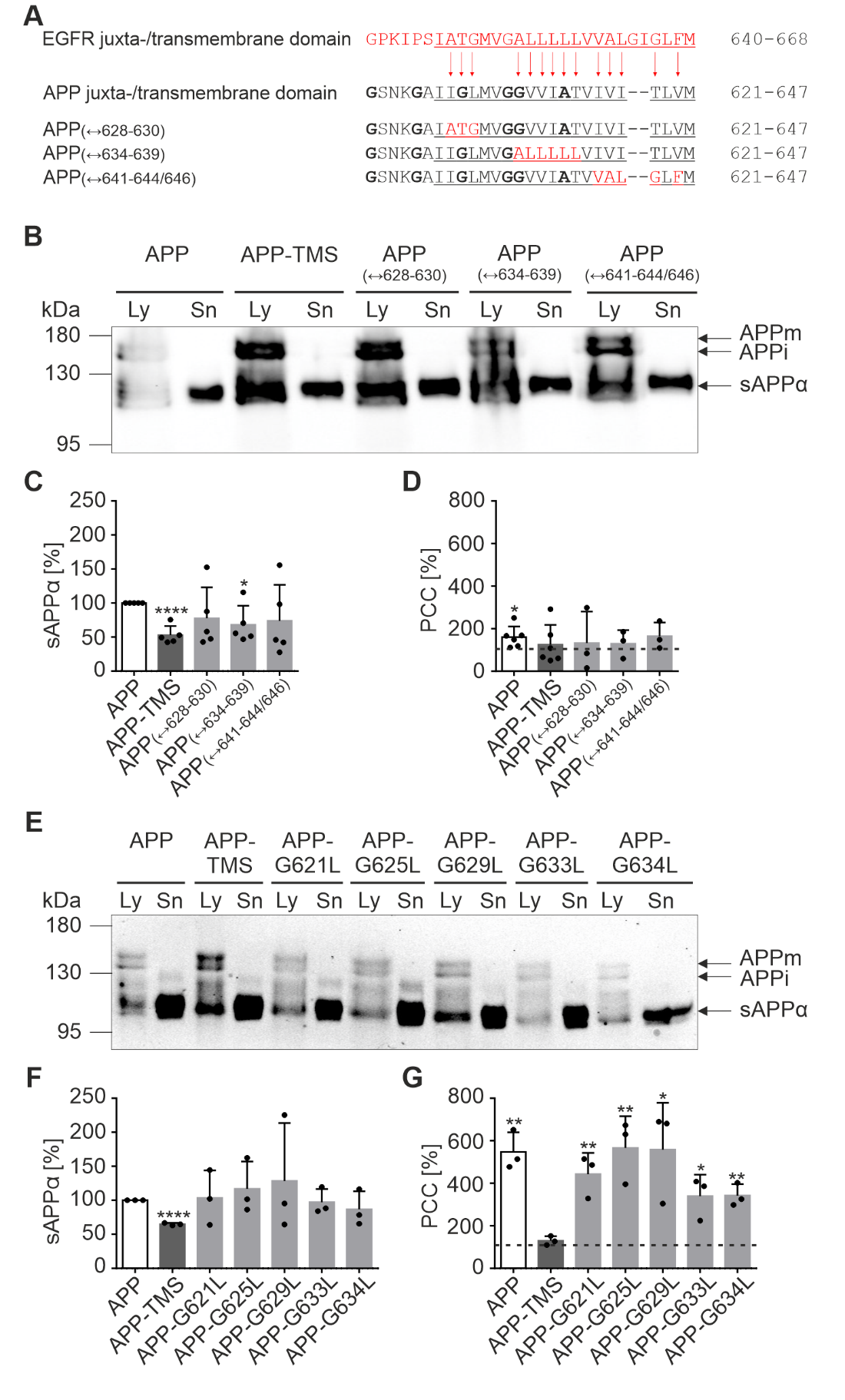


**Figure S8** *Effect of modifications of the juxta- and transmembrane domain on α-processing and crosslinking with ADAM10.* (A) Amino acid sequences of the juxta- and transmembrane segment of the EGFR (aa 640-668) and APP (aa 621-647). Compared to the TMS (underlined) of EGFR, the TMS of APP (underlined) lacks two amino acids and differs at position 628-630, 634-639, 641-644 and 646 (see red arrows and amino acids depicted in red). Additionally, APP contains three GxxxG motifs (marked in bold letters G) and one GxxxG-like motif (marked in bold letters G and A). APP(↔628-630) carries the mutations I628A, G629T, L630G, APP(↔634-639) the mutations G634A, V635L, V636L, I637L, A638L, T639L, and APP(↔641-644/646) the mutations I641V, V642A, I643L, T644G, V646F. (B, E) In one series, HepG2 cells are transfected with GFP-tagged APP/APP-TMS in parallel with (B) APP(↔628-630), APP(↔634-639) and APP(↔641-644/646) and in another series along with (E) APP-G621L, APP-G625L, APP-G629L, APP-G33L or APP-G634L. They were grown in the presence of 10 µM DAPT and lysed 21 h after transfection. Lysate (Ly) and supernatant (Sn) are analyzed by Western blot. (C, F) The sAPPα band intensities are normalized to the sum of immature (APPi) and mature APP (APPm) band intensities, and related to the APP value, which is set to 100 %. Values are given as means ± SD ((B, C) n = 5 experiments; (E, F) n = 3 experiments). (D, G) Pearson Correlation Coefficient (PCC) between ADAM10 and APP constructs as indicated. Analysis as in **Fig. 5B**, showing only the CoP condition. For example images see **Fig. S9** (for D) or **Fig. S10** (for G). (D, G) Values are given as means ± SD (n = 3 - 6 experiments, 15 - 20 membrane sheets per experiment and condition. Unpaired Student’s t-tests compare (C, F) APP-mutants to APP or (D, G) CoP to fixed (****p <0.0001; ***p <0.001; **p <0.01; *p <0.05).


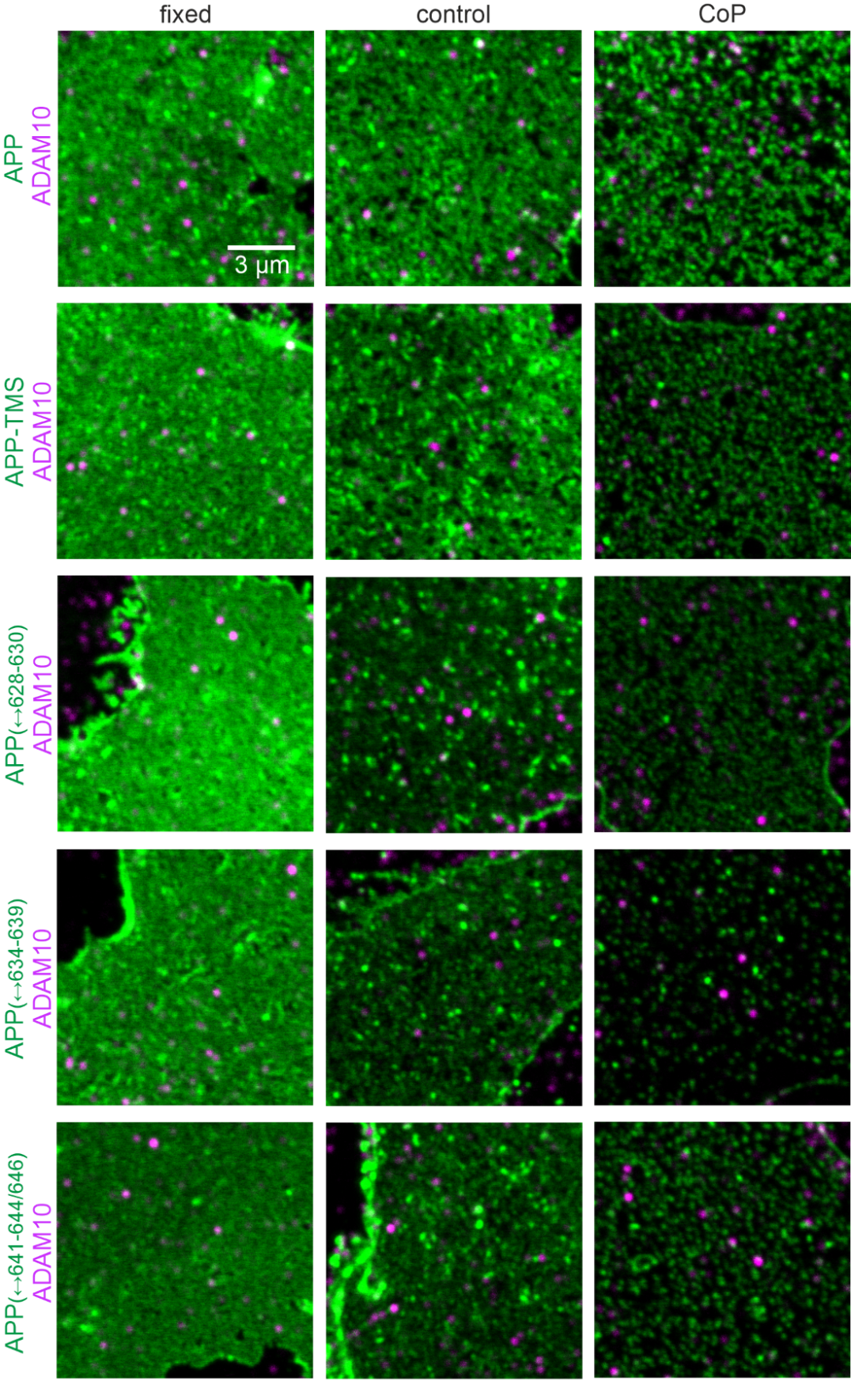


**Figure S9** *Crosslinking of GFP-tagged APP with modifications of the transmembrane segment.*

Confocal micrographs of membrane sheets generated from HepG2 cells expressing the indicated constructs, either directly fixed (*left column*), or incubated without (control; *middle column*) or with crosslinking antibodies (CoP; *right column*), followed by immunostaining for ADAM10. GFP-tag (green), ADAM10 immunostaining (magenta). For analysis of the PCC see **Fig. S8D**.


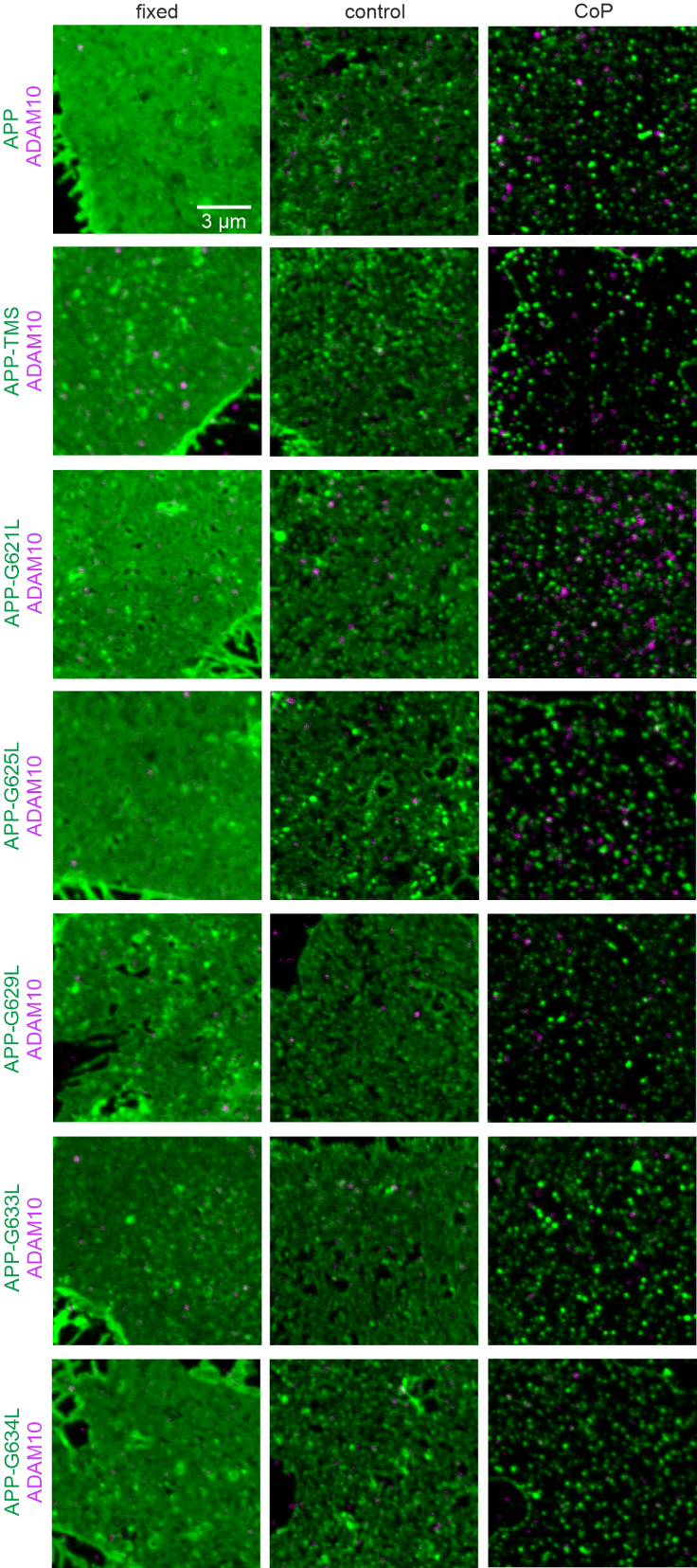


**Figure S10** *Crosslinking of GFP-tagged APP with modifications of the transmembrane segment.*

Confocal micrographs of membrane sheets generated from HepG2 cells expressing the indicated constructs, either directly fixed (*left column*), or incubated without (control; *middle column*) or with crosslinking antibodies (CoP; *right column*), followed by immunostaining for ADAM10. GFP-tag (green), ADAM10 immunostaining (magenta). For analysis of the PCC see **Fig. S8G**.


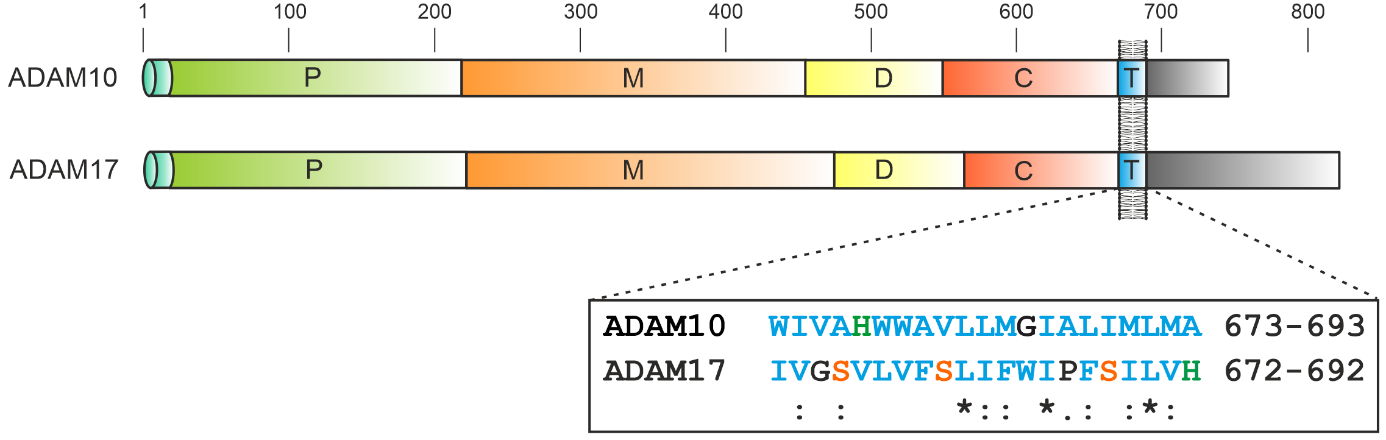


**Figure S11** *Domain structure of human ADAM10 and ADAM17*

The secretase domain structure is a signal peptide (cyan) followed by a pro (P; green), a metalloprotease (M; orange), disintegrin (D; yellow), cysteine-rich (C; red), transmembrane (T; blue) and cytoplasmic domain (gray). Box, alignment of the amino acid sequences of the TMS using the Clustal Omega alignment tool (52). Blue, hydrophobic amino acid residues; orange, polar uncharged; green, positively charged; black, other residues; (*), fully conserved residues; (:), strong conservation scoring > 0.5 in the Gonnet PAM 250 matrix (53); (.), weak conservation scoring ≤ 0.5 and > 0 in the Gonnet PAM 250 matrix.


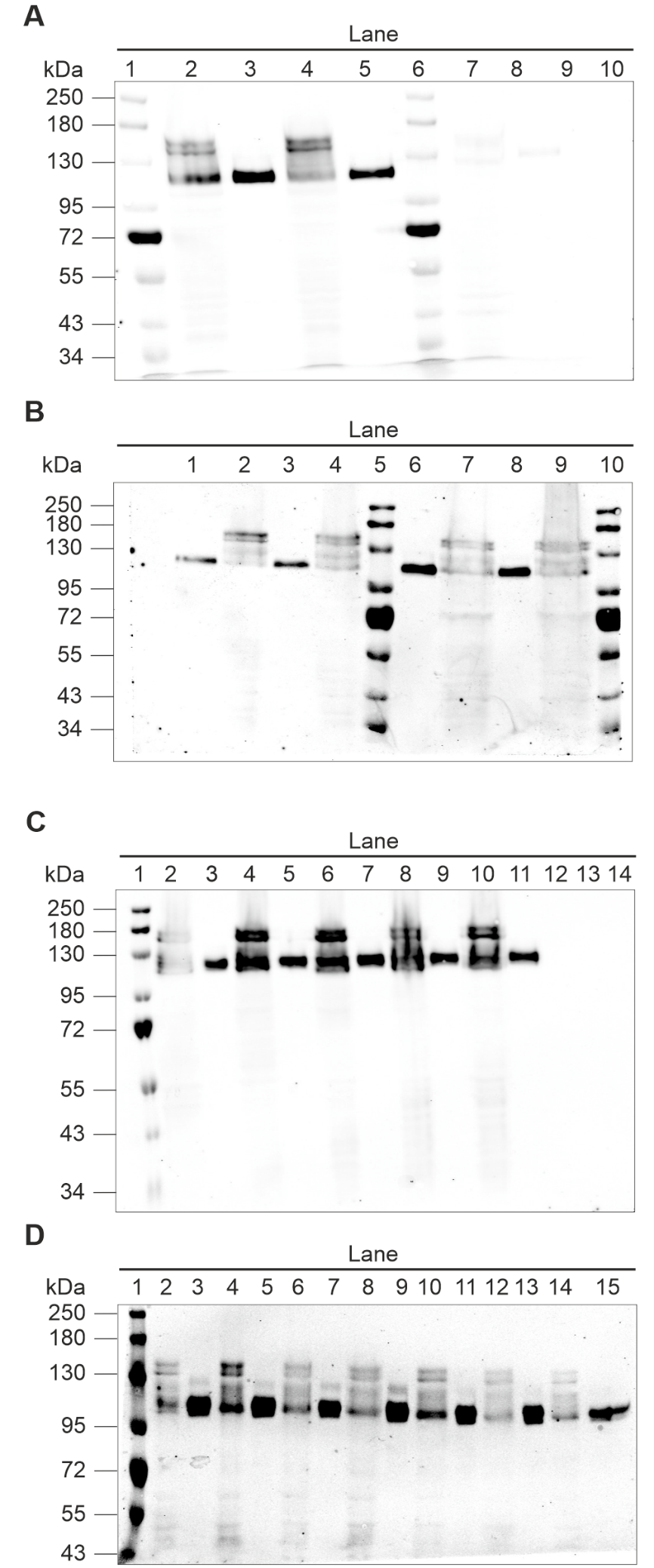


**Figure S12** *The full-length images of Western blots*.

(A) The full-length Western blot for **Fig. 5D**. Lane 1 and lane 6 (protein marker; cat#P7719, New England Biolabs). Lane 2 (APP, Ly), lane 3 (APP, Sn), lane 4 (APP-TMS, Ly) and lane 5 (APP-TMS, Sn) were cropped and used for the figure. (B) The full-length Western blot for Fig. **S4A**. Lane 5 and lane 10 (protein marker; cat#P7719, New England Biolabs). Lane 1 (control, Sn), lane 2 (control, Ly), lane 3 (+ PMA, Sn) and lane 4 (+ PMA, Ly) were cropped and used for the figure. (C) The full-length Western blot for **Fig. S8B**. Lane 1 (protein marker; cat#P7719, New England Biolabs). Lane 2 (APP, Ly), lane 3 (APP, Sn), lane 4 (APP-TMS, Ly), lane 5 (APP-TMS, Sn), lane 6 (APP(↔628-630), Ly), lane 7 (APP(↔628-630), Sn), lane 8 (APP(↔634-639), Ly), lane 9 (APP(↔634-639), Sn), lane 10 (APP(↔641-644/646), Ly) and lane 11 (APP(↔641-644/646), Sn) were cropped and used for the figure. (D) The full-length Western blot for **Fig. S8E**. Lane 1 (protein marker; cat#P7719, New England Biolabs). Lane 2 (APP, Ly), lane 3 (APP, Sn), lane 4 (APP-TMS, Ly), lane 5 (APP-TMS, Sn), lane 6 (APP-G621L, Ly), lane 7 (APP-G621L, Sn), lane 8 (APP-G625L, Ly), lane 9 (APP-G625L, Sn), lane 10 (APP-G629L, Ly), lane 11 (APP-G629L, Sn), lane 12 (APP-G633L, Ly), lane 13 (APP-G633L, Sn), lane 14 (APP-G634L, Ly) and lane 15 (APP-G634L, Sn) were cropped and used for the figure.
